# An Interactive Text Message Intervention to Reduce Binge Drinking in Young Adults: A Randomized Controlled Trial with 9-Month Outcomes

**DOI:** 10.1371/journal.pone.0142877

**Published:** 2015-11-18

**Authors:** Brian Suffoletto, Jeffrey Kristan, Tammy Chung, Kwonho Jeong, Anthony Fabio, Peter Monti, Duncan B. Clark

**Affiliations:** 1 Department of Emergency Medicine, University of Pittsburgh School of Medicine, Pittsburgh, Pennsylvania, United States of America; 2 Department of Psychiatry, University of Pittsburgh, Pittsburgh, Pennsylvania, United States of America; 3 Department of Epidemiology, Graduate School of Public Health, University of Pittsburgh, Pittsburgh, Pennsylvania, United States of America; 4 Center for Alcohol and Addiction Studies, Brown University, Providence, Rhode Island, United States of America; Centre for Addiction and Mental Health, CANADA

## Abstract

**Background:**

Binge drinking is associated with numerous negative consequences. The prevalence and intensity of binge drinking is highest among young adults. This randomized trial tested the efficacy of a 12-week interactive text message intervention to reduce binge drinking up to 6 months after intervention completion among young adults.

**Methods and Findings:**

Young adult participants (18–25 y; n = 765) drinking above the low-risk limits (AUDIT-C score >3/4 women/men), but not seeking alcohol treatment, were enrolled from 4 Emergency Departments (EDs) in Pittsburgh, PA. Participants were randomized to one of three conditions in a 2:1:1 allocation ratio: SMS Assessments + Feedback (SA+F), SMS Assessments (SA), or control. For 12 weeks, SA+F participants received texts each Thursday querying weekend drinking plans and prompting drinking limit goal commitment and each Sunday querying weekend drinking quantity. SA+F participants received tailored feedback based on their text responses. To contrast the effects of SA+F with self-monitoring, SA participants received texts on Sundays querying drinking quantity, but did not receive alcohol-specific feedback. The control arm received standard care. Follow-up outcome data collected through web-based surveys were provided by 78% of participants at 3- months, 63% at 6-months and 55% at 9-months. Multiple imputation-derived, intent-to-treat models were used for primary analysis. At 9-months, participants in the SA+F group reported greater reductions in the number of binge drinking days than participants in the control group (incident rate ratio [IRR] 0.69; 95% CI .59 to.79), lower binge drinking prevalence (odds ratio [OR] 0.52; 95% CI 0.26 to 0.98]), less drinks per drinking day (beta -.62; 95% CI -1.10 to -0.15) and lower alcohol-related injury prevalence (OR 0.42; 95% CI 0.21 to 0.88). Participants in the SA group did not reduce drinking or alcohol-related injury relative to controls. Findings were similar using complete case analyses.

**Conclusions:**

An interactive text-message intervention was more effective than self-monitoring or controls in reducing alcohol consumption and alcohol-related injury prevalence up to 6 months after intervention completion. These findings, if replicated, suggest a scalable approach to help achieve sustained reductions in binge drinking and accompanying injuries among young adults.

**Trial Registration:**

ClinicalTrials.gov NCT01688245

## Introduction

Young adulthood is a period of developmental transition when behavioral patterns related to substance use, especially alcohol consumption, can peak. For example, almost half of US college students report binge drinking (defined as the consumption of four or more drinks per occasion for a woman and five or more drinks for a man) in the prior 2 weeks [1]. Binge drinking is strongly associated with alcohol-related injuries [2] and increased risk for the onset of alcohol use disorders [3].

The emergency department (ED) is commonly used by young adults for primary care [4], and can be an opportune point of contact to link young adults with alcohol misuse to effective prevention resources. Brief in-person interventions in the ED aimed at preventing binge drinking and alcohol-related injuries in young adults have been shown in prior research to be effective [5,6], but have not successfully scaled to have population-level impact [7]. This may be due to numerous barriers, most obvious being the cost and resources needed to provide one-on-one counseling [8].

One way to overcome these barriers is by delivering computerized interventions through the internet and mobile devices. Computerized delivery enables standardization of support materials, reduced stigma relative to in-person reporting, and economies of scale. A meta-analysis of computer-delivered alcohol interventions for college students suggests that they can produce short-term reductions in alcohol consumption, but may not be as effective as face-to-face interventions [9]. Investigators have begun to explore ways to improve the effectiveness of computerized interventions for alcohol misuse, including the use of mobile phones [10]. Mobile phones can reach individuals in the context of the real world where health behaviors are challenged and frequently fail [11].

The ubiquity of mobile phone ownership worldwide [12] and frequency of text messaging (short message service: SMS) for routine communication [13] offer an unprecedented opportunity to explore SMS as a modality for delivering computerized interventions in the context of daily life. Our group recently reported that young adult patients who screened positive for past hazardous drinking in the ED and randomized to an SMS intervention reported fewer binge drinking days than SMS self-monitoring or control during the 12-week period of SMS intervention exposure [14]. Still, it remains unknown whether exposure to an SMS intervention can result in sustained reductions in binge drinking or has any effect on alcohol-related injury. Therefore, the aim of this study was to examine the durability of SMS intervention effects up to 6-months post-intervention completion. Although the intervention does not aim to reduce alcohol-related risk behaviors specifically, we explored SMS intervention effects on drinking-related injury prevalence over follow-up.

## Methods

A randomized, controlled, blindly evaluated intervention trial was used to determine differential effects of SMS Assessments + Feedback intervention (SA+F) versus SMS Assessments (SA) versus no-SMS (control) on self-reported alcohol consumption and alcohol-related injuries in young adults. This study was registered with clinicaltrials.gov (NCT01688245). Study procedures were approved by the Institutional Review Board of the University of Pittsburgh. The trial has been described previously [15]. The CONSORT requirements were followed (S1 CONSORT Checklist).

### Participants

Patients aged 18–25 years who presented to one of 4 EDs in Pittsburgh, PA were eligible to participate if they (1) were medically stable, (2) spoke English, (3) were not seeking treatment for alcohol or drugs, and (4) reported past hazardous drinking based on an AUDIT-C score >3 for women and >4 for men [16]. Patients were excluded if they reported (1) not owning a personal mobile phone with text messaging, (2) past treatment for drug or alcohol disorder, (3) current treatment for psychiatric disorder, or (4) current enrollment in high school. We did not screen or enroll patients with acute alcohol intoxication because it would not be possible to obtain informed consent. All patients with a positive AUDIT-C screen received brief, standard alcohol risk-reduction advice.

### Design and procedures

#### Baseline assessment

If eligible, patients provided written informed consent and completed a web-based baseline assessment in the ED. Contact information was collected and participants were reimbursed for their time (US$10). The baseline assessment collected information on demographic characteristics, alcohol use over the past 30 days, other substance use and number of injuries during the past 3 months. We took steps to minimize reporting bias by using a self-guided web-based entry system, and asking friends and family to leave the room during the assessment. We also asked the treating ED physician whether or not they felt that the care encounter was related to alcohol.

#### Randomization

Participants who completed the baseline survey were randomly assigned to one of three groups: SA+F, SA, and control in a 2:1:1 ratio to ensure enough SA+F participants to examine mechanisms of change. Randomization was generated in blocks of eight for each recruitment site by a computer-generated algorithm and allocated electronically. Participants were not told to which group they were randomized to minimize expectation bias. Research associates were also blind to treatment allocation.

#### SMS intervention (SA+F) group

Participants in SA+F engaged in brief two-way text message dialogue sessions each Thursday and Sunday for 12-weeks. SA+F aims to increase awareness of weekend drinking intentions and behavior and increase goal-striving and goal-attainment toward reduced alcohol consumption. The intervention is largely based on the Theory of Planned Behavior [17], refined through pilot research [18] and young adult input [19]. Each Thursday (proximal to typical binge drinking days) [20], SMS queries assessed whether the individual had a weekend drinking plan. If a plan to drink was reported, SMS queried whether the person was willing to set a goal to limit drinking below the threshold of 4 drinks for women (5 for men) per drinking occasion over that weekend. Based on the individual’s response, they received tailored feedback messages aimed at increasing motivation toward reduced alcohol consumption. Each Sunday (to reduce bias in recall of weekend drinking behavior) [21], SMS queries assessed the largest number of drinks that an individual consumed on any occasion that weekend. Based on the individual’s response, they received tailored feedback messages that supported low weekend alcohol consumption or aimed to encourage reflection on their alcohol consumption. A more detailed description of this structured intervention is reported elsewhere [14,15].

#### SMS Assessments (SA) group

Participants in the SA group did not receive any pre-weekend (Thursday) SMS queries, but received SMS drinking queries each Sunday for 12-weeks that were identical to SA+F, without receiving any alcohol-related feedback. The SA group is critical to separate the effect of SA+F from that associated with potential drinking assessment reactivity that may result from asking participants to self-monitor and report their alcohol consumption each week for 12 weeks [22].

#### No-SMS (control) group

Participants in the no-SMS control condition did not participate in any text messaging related to alcohol use.

#### Follow-up assessment

Three-, 6- and 9-months after enrollment in the ED, all participants were asked to complete follow-up surveys by logging in to a password-protected website. Participants were notified by SMS to access the survey web site. Those who did not complete their web-based follow-up within 1 week of follow-up date were contacted up to 6 times through email, text message and phone calls. Participants were reimbursed for completing the follow-ups (US$20 at 3-month, US$30 at 6-months, US$40 at 9-months).

#### Measures

Demographics included age, sex, race, ethnicity, highest education level, living arrangement, and employment status. Substance use over the past 3 months was assessed using the NIDA Modified Alcohol, Smoking and Substance Involvement Screening Test (NM-ASSIST) [23]. Alcohol use was assessed using the Timeline Follow Back (TLFB) method [24], where participants entered the number of drinks consumed each day in a calendar covering the past 30 days. Memory aids were used to enhance recall (e.g., visual calendar with key dates and holidays to serve as anchors for reporting drinking; a visual chart of standard drink sizes to reduce variability in quantity). Alcohol-related injuries were assessed using the revised Injury Behavior Checklist (IBC) [25,26]. Participants recorded the number of times each of 18 injuries occurred during the past 3 months, including a question on whether they were injured by being in a physical fight with someone. For each injury, the patient also recorded whether he or she was drinking within 2 hours of the injury.

### Outcomes

Primary outcomes include the number of self-reported binge drinking days (4+/5+ drinks for females/males; continuous) and binge drinking prevalence (yes/no) over the past 30 days. Secondary outcomes include drinks per drinking day (continuous) over the past 30 days and alcohol-related injury prevalence (yes/no) over the past 3 months. All alcohol consumption outcomes were calculated using the TLFB. All alcohol-related injury outcomes were calculated using the IBC. Outcomes were measured at four time points: baseline, three, six and nine months.

### Sample size calculation

Data from a previous pilot trial in the same setting and the same population were used to approximate drinking outcomes [18], while data from publications in the field gave estimates of attrition rates [26]. Sample size calculations were based on an estimated mean change in number of binge drinking days from baseline to 9-months of -2.2 (SD 5.4) in SA+F and -0.7 (SD 4.1) in controls. To detect a difference between these means with 90% power using a two-sided t-test with unequal variances at 1% significance level, and allowing for at least 30% attrition, an a priori sample size of 750 (2:1:1 ratio across conditions) was chosen.

### Analysis

All analyses were conducted using STATA statistical software, version 12.1. An intent-to-treat analysis was conducted by including all participants who completed baseline assessments and who were randomized to intervention conditions, regardless of the amount of SMS dialogue they completed. Because of the non-trivial amount of missing outcome data, and because outcome data was missing at random (measured covariates associated with missingness), we used multiple imputation procedures to minimize bias during the estimation of standard errors [27]. Imputation models were as follows: for number of binge drinking days, we used a Poisson distribution model; for any binge drinking day, we used a logit distribution model; for drinks per drinking day, we used a regression distribution model; for any alcohol-related injury, we used a logit distribution model. Predictors in the models included sex, baseline drinking severity (AUDIT-C score), race, college enrollment, and past 30-day drinking (from prior time points). The final inference was combined from 50 sets of imputed data, as per recommendations [28].

Given the longitudinal and repeated nature of the outcomes, we transformed the data from wide to long and designated it as panel data. Because the outcomes are correlated across time periods, we used population-average models, i.e. generalized estimating equations (GEE) to estimate the average impact of the intervention over time. Because we did not want to impose any constraints on the outcome data models, we used an unstructured covariance matrix. Specifically, a Poisson regression model was used for number of binge drinking days, a logit regression model was used for any binge drinking days and any alcohol-related injury, and a linear regression model was used for drinks per drinking day. A treatment by time interaction was included in the models to display the effect of the intervention over time with respect to each outcome. Models controlled for covariates that were potentially associated with outcomes, including sex, age, race, college enrollment, and enrollment site. To ensure that imputation estimates were not biased, we then performed Listwise deletion (i.e., using complete cases only). To understand how the SMS intervention differs from self-monitoring, the SA+F and SA groups were both compared to the control group. Estimated treatment effects are reported with 95% confidence intervals.

## Results

### Recruitment and retention

**Fig 1:**
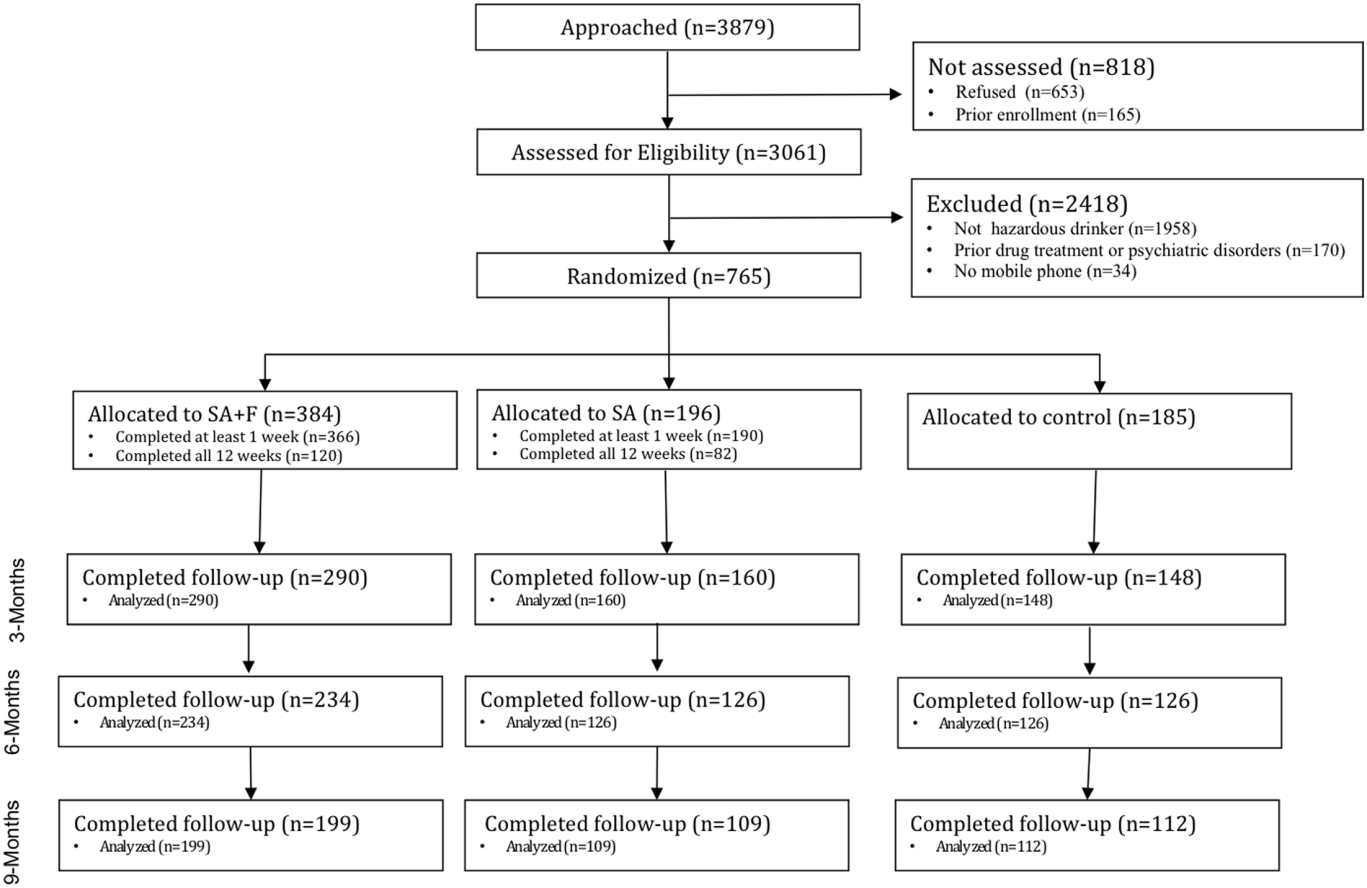
CONSORT Diagram. SA + F, SMS Assessments + Feedback intervention; SA, SMS Assessments.

Between November 1, 2012 and November 5, 2013, research associates screened 3,061 potentially eligible young adult patients, and stopped recruitment after enrolling the target sample of 765 (*n = 384* in SA+F; *n = 196* in SA; *n = 185* in control) (Fig 1). The sample was diverse in terms of sex (65% women), race (43% black), and education (56% not college educated) (Table 1). Average age was 22.0 (SD = 2.0), with 26% underage drinkers (<21 years of age). Only 2% of those enrolled presented to the ED for alcohol-related illness or injury. There were no differences in the baseline characteristics for the three randomized groups using chi-squared tests for categorical variables and analysis of variance tests for continuous variables.

**Table 1:** Baseline sample characteristics.

Characteristics	SA+F (n = 384)	SA (n = 196)	Control (n = 185)
**Age, mean (SD), y**	22.0 (2.0)	22.0 (2.0)	21.8 (2.1)
**Female**	251 (65.4)	125 (63.8)	124 (67.0)
**Race**			
Black	158 (41.2)	88 (44.9)	83 (44.9)
White	190 (49.5)	98 (50.0)	88 (47.6)
Other	36 (9.4)	10 (5.1)	14 (7.6)
**Hispanic Ethnicity**	22 (5.7)	10 (5.1)	15 (8.1)
**Current College enrollment**	162 (42.2)	85 (43.4)	87 (47.0)
**Employment**			
Not working	120 (31.2)	62 (31.6)	61 (33.0)
Part-time	110 (28.7)	59 (30.1)	62 (33.5)
Full-time	154 (40.1)	75 (38.3)	62 (33.5)
**Other Substance Use last 3 months**			
Daily or almost daily tobacco	145 (37.8)	72 (36.7)	64 (34.6)
Any cannabis	197 (51.3)	94 (50.0)	95 (51.4)
**AUDIT-C score, mean (SD)**	6.3 (2.2)	6.2 (2.1)	6.3 (2.2)
**ED Visit Due to Alcohol**	12 (3.1)	3 (1.5)	4 (2.2)

SA+F, SMS Assessments + Feedback; SA,SMS Assessments; SD, standard deviation; AUDIT-C, Alcohol Use Disorders Identification Test for Consumption; ED, Emergency Department.

All data presented as number (%) unless otherwise specified.

Replies to text message queries were high overall in SA+F and SA groups, but decreased similarly across groups from week 1 to 12. Specifically, in week 1 90.9% of SA+F and 93.3% of SA responded to Sunday drinking quantity query, and in week 12 66.4% of SA+F and 72.8% of SA responded to Sunday drinking quantity query. Roughly one-third (33%) of participants in SA+F and SA groups completed all text queries.

Follow-up retention was 78.2% (n = 598) at 3-months, 63.5% (n = 486) at 6-months and 54.9% (n = 420) at 9-months, with no statistically significant differences by study group using chi-squared tests. Compared to participants who completed follow-up at 9-months, those lost to follow-up were more likely to self-identify as being of black race (38.1% vs. 55.1%; p<0.0001), less likely to be currently enrolled in college (51.7% vs. 33.9% p<0.0001), and with higher baseline AUDIT-C scores (mean 6.03 vs. 6.57; p = .0005).

### Intervention effects

**Fig 2:**
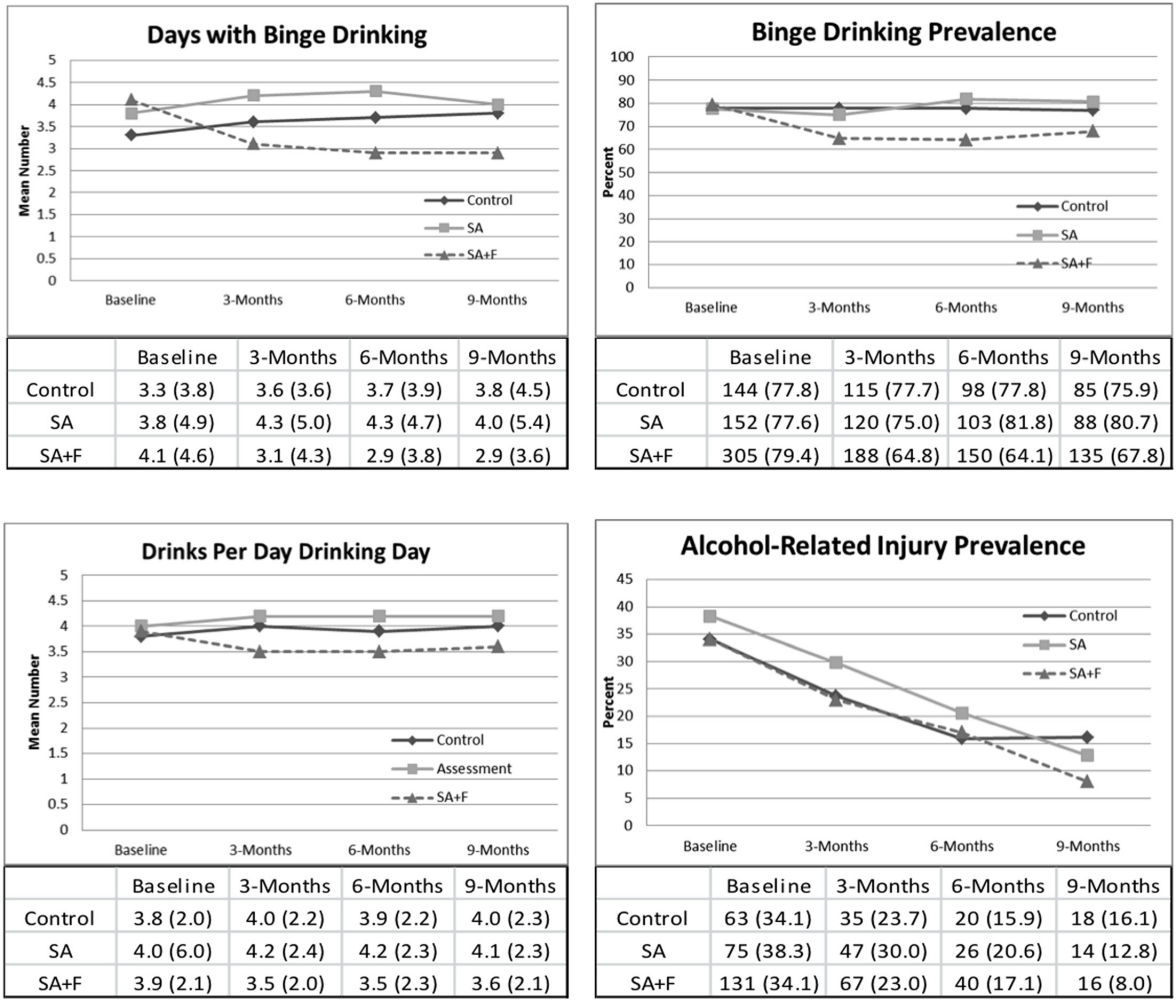
Self-reported alcohol consumption and alcohol-related injuries at 3-, 6- and 9-months follow-ups.

Self-reported alcohol consumption and alcohol-related injury at baseline, 3-, 6- and 9-months after enrollment appear in Fig 2. Longitudinal analysis is presented in Table 2. There was a significant intervention by time interaction at 3-, 6- and 9-months, with SA+F participants reporting less drinking across all measured alcohol consumption outcomes when compared to control participants. There was a significant time by intervention interaction only at 9-months for alcohol-related injury prevalence, with SA+F participants reporting lower alcohol-related injury prevalence than control participants. There were no significant reductions in alcohol-related outcomes when comparing SA participants to control participants. Findings were similar using complete case analyses.

**Table 2:** Longitudinal analysis of outcome measures from baseline to 9-months.

	Number of binge drinking days^a^	Binge drinking prevalence^b^	Drinks per drinking day^c^	Alcohol-related injury prevalence^b^
	*IRR (95% CI)*	*OR (95% CI)*	*beta (95% CI)*	*OR (95% CI)*
Treatment (reference = control)				
SA	1.10 (0.98; 1.22)	1.01 (0.61; 1.70)	0.19 (-0.20; 0.59)	1.20 (0.78; 1.84)
SA+F	**1.15 (1.04; 1.26)**	1.09 (0.69; 1.71)	0.06 (-0.28; 0.41)	0.96 (0.66; 1.41)
Time (reference = baseline)				
3 months	**1.13 (1.04; 1.24)**	0.93 (0.57; 1.51)	0.31 (-0.03; 0.64)	0.60 (0.78; 1.84)
6 months	**1.19 (1.08; 1.32)**	0.93 (0.55; 1.57)	0.30 (-0.08; 0.67)	**0.39 (0.23; 0.65)**
9 months	**1.20 (1.08; 1.34)**	0.87 (0.52; 1.47)	0.35 (-0.04; 0.75)	**0.41 (0.23; 0.71)**
Treatment x Time				
3 months x SA	1.07 (0.85, 1.21)	0.90 (0.46; 1.77)	-0.21 (-0.68; 0.26)	1.14 (0.63; 2.08)
6 months x SA	1.04 (0.91; 1.18)	1.31 (0.62; 1.78)	-0.29 (-0.81; 0.23)	1.10 (0.54; 2.16)
9 months x SA	0.96 (0.83; 1.11)	1.29 (0.62; 2.70)	-0.35 (-0.93; 0.23)	0.56 (0.24; 1.28)
3 months x SA+F	**0.75 (0.67; 0.84)**	**0.47 (0.26; 0.84)**	**-0.64 (-1.10; -0.23)**	0.93 (0.54; 1.61)
6 months x SA+F	**0.70 (0.62; 0.79)**	**0.42 (0.23; 0.79)**	**-0.72 (-1.18; -0.26)**	1.10 (0.55; 2.03)
9 months x SA+F	**0.69 (0.60; 0.79)**	**0.52 (0.26; 0.98)**	**-0.62 (-1.10; -0.15)**	**0.42 (0.21; 0.88)**

SA+F,SMS Assessments + Feedback; SA,SMS Assessments; OR, Odds Ratio; CI, Confidence Interval; IRR, Incident Rate Ratio.

^a^Population-averaged Poisson regression model.

^b^ Population-averaged logit regression model.

^c^Population-averaged linear regression model.

All models adjusted for sex, age, race, college enrollment, and enrollment site.

SA+F, SMS Assessments + Feedback; SA = SMS Assessments.

Tables under graphs of binge drinking days and drinks per drinking day presented as mean (standard deviation). Tables under graphs of participants with any binge drinking day and any alcohol-related injury presented as number of participants (%).

## Discussion

This randomized trial provides the first experimental evidence that an automated and interactive text-message intervention can produce sustained reductions in alcohol consumption in a diverse sample of young adults. On average, young adults exposed to the SMS intervention reported one less binge drinking day per month and there was a decrease of around 12% in the proportion of young adults reporting any binge drinking days up to 6-months after completing the intervention. Excessive alcohol consumption, including binge drinking, costs the United States $223.5 billion in 2006 from losses in productivity, health care, crime, and other expenses [29]. On average, 6 people die every day from alcohol poisoning in the US [30]. We estimate that the 13 young adults need to be exposed to the SMS intervention to prevent one young adult from binge drinking. Given the low cost to send text messages and the capacity to deliver them to almost every young adult in the US, an SMS intervention targeting binge drinking could have public health impact on reducing both immediate and long-term health problems.

Compared to in-person ED brief interventions tested in large randomized trials, where effects diminish over time [31] or were not more effective in reducing alcohol consumption in young adults compared to controls [32], the SMS intervention we tested accomplished both tasks. The mean effect size estimated in this study (Cohen's d = 0.13) was comparable to other computerized interventions for reducing college student binge drinking (Cohen's d = 0.10) [33]. A recent meta-analysis of 14 studies examining SMS interventions for substance use among young adults reported an effect size of 0.25 [34], however 12/14 of the studies addressed smoking (not drinking) and enrolled treatment-seeking individuals, making comparison with this study less applicable.

The intervention used a computer program and SMS technology to conduct text dialogue, providing an efficient and low-cost way to systematically maintain contact with young adults over time. The relatively high fidelity to SMS queries on Thursday and Sunday over 12 weeks suggests that asynchronous and electronically-mediated communication may be more acceptable to young adults than phone call boosters [35]. The feedback messages were based on decision rules that were developed prior to trial initiation, essentially eliminating the uncontrolled variability that exists in delivery of in-person alcohol interventions [36]. By interacting with individuals in the context of their lives, SMS messages can provide a “cue to action” when self-regulation processes are most vulnerable [37].

We did not find any reduction in alcohol consumption measures in the SA group compared to the control. This contradicts the literature showing an association between alcohol assessments and drinking behavior [22] and a review of 42 behavior change techniques used in brief interventions for alcohol use showing that the single most influential technique was prompting self-recording of alcohol intake [38]. Our finding suggests that in our sample of young adults, self-monitoring of weekend drinking alone was not effective at reducing alcohol consumption. Given that the frequency of SMS contact in the SA group did not match the SA+F group, we cannot comment on whether the lack of drinking reductions in SA participants may have been due to lower frequency of SMS contact.

### Study limitations

These trial findings are most limited by the relatively high rate of attrition over follow-up, where estimates of effect could be vulnerable to bias. Given that intervention condition was not differentially associated with attrition, we feel that the biases are likely to be equal across groups. Also, this lost-to-follow-up rate are similar to other substance use trials recruiting patients from the ED [26,31]. The outcome measures were based on self-reported data, which may be subject to recall or social desirability biases, and may have increased the apparent efficacy of the intervention. However, inclusion of an SMS assessment group (SA), which did not show a significant change in drinking over follow-up, helped to guard against this possibility. Although a diverse sample of young adults were recruited, our results only apply to those who screen positive for hazardous alcohol use in the Emergency Department and are not seeking help to reduce their drinking. Future trials could assess whether the intervention can also benefit different age groups (i.e. older adolescents, older adults), those identified in other settings (i.e. online) or those seeking help with reducing their drinking. Only 2% of our enrolled population presented to the ED with an alcohol-related injury or illness, precluding us from determining whether the intervention would be effective in this subgroup.

### Unanswered questions and future research

Future trials need to determine the “active ingredients” of SMS alcohol misuse prevention interventions (i.e. self-monitoring, goal setting prompts and feedback) on alcohol outcomes. Future research should also explore the relationship between changes in alcohol consumption and alcohol-related injuries more closely. Qualitative research will provide further valuable insights into how young adults feel about SMS interventions and how they can be improved to increase engagement and saliency. Finally, studies on cost-effectiveness need to be conducted before this or similar automated mobile communication programs are implemented on a larger scale in routine health care or included in insurance reimbursement programs.

## Supporting Information

S1 CONSORT Checklist (DOC)

S1 Protocol IRB Protocol. (DOCX)
