# Supplementary material for: An Interactive Text Message Intervention to Reduce Binge Drinking in Young Adults: A Randomized Controlled Trial with 9-Month Outcomes
Source: PLoS One. 2015 Nov 18;10(11):e0142877. doi: 10.1371/journal.pone.0142877 (PMC4651466; doi:10.1371/journal.pone.0142877)
Supplement: S1 Protocol — (DOCX) [file pone.0142877.s002.docx]

Provide a short title for this study (200 characters or less):

Texting to Reduce Alcohol Consumption (TRAC) Trial

T1.0 Select the type of application:

New Research Study

T2.0 Is the proposed research study limited to the inclusion of deceased individuals?

*no

The review and approval of proposed innovative practices are not subject to IRB review and approval. The introduction of innovative procedures or therapies into clinical practice (i.e., independent of a research activity approved by the IRB) should be reviewed with the applicable department chairperson and the UPMC Technology Assessment Committee/Innovative Practices Sub-Committee prior to their implementation. The contact person is Mary Gardner at 412-647-6883.

T2.1

Are any research activities being conducted at the VA Pittsburgh Healthcare System or with VA funds?

*no

Respond to the following questions to determine the IRB-of-record:

Research is conducted using only VA records and/or subjects recruited thru the VA:

University or UPMC facilities are not engaged in research:

University or UPMC funds are not expended in direct support of research:

If all true, then the VA is the IRB-of-record and UPitt IRB review is not required.

If all false, only UPitt IRB review is required.

Otherwise, dual review from both the VA and UPitt IRB is required.

Read carefully- Studies are not eligible for NCI Central IRB review if any of the following are required:

• review by the Institutional Biosafety Committee (IBC)

• waiver of HIPAA authorization

• conduct of any research procedures at a site outside of the Commonwealth of Pennsylvania

• enrolling prisoners

Please select the external IRB of record:

Quality assurance projects are not subject to IRB review and approval. UPMC has adopted an oversight process that requires the submission of all quality assurance projects for review. At UPMC, submissions are reviewed by the Total Quality Council. The contact person is Juliet Jegasothy at 412-612-3304.

Research studies that are limited to the inclusion of deceased individuals are not subject to IRB review and approval. Research performed on individuals who have been declared legally dead and/or research involving the collection of tissues from deceased individuals is not subject prior review and approval by the University of Pittsburgh IRB.

There are, however, ethical issues associated with research conducted on or involving deceased individuals. To address these ethical issues, all University faculty who desire to perform research on or involving deceased individuals must submit a project application for review and approval by the Committee for Oversight of Research and Clinical Training Involving the Dead Research Involving the Dead (CORID). Note that, as per UPMC policies, research involving the medical records of deceased individuals is subject to obtaining the written consent of the decedents’ next-of-kin or the executors of the decedents’ estates.

For studies that include BOTH living and deceased subjects, IRB review and approval is required.

Emergency Use is the use of a test article on a human subject in a life-threatening situation in which no standard acceptable treatment is available, and in which there is not sufficient time to obtain IRB approval [21 CFR312.310]. Detailed information on the submission process is available on the IRB website under the A-Z Guidance, Emergency Use.

All of the following conditions must exist to justify the emergency use of an unapproved investigational drug, biologic, or device. Check all the boxes that apply:

Selections

There are no items to display

[reviewer notes¬]

Triage Section

T3.0 What is the anticipated risk to the research participants?

Minimal Risk

T3.1 Why do you feel that all aspects of this research study, including screening and follow-up, involve no more than minimal risk to the research subjects?

Screening entails asking questions regarding alcohol use used in routine care.

Research procedures exposes participants to routine survey questions through password-protected web-site and text-messaging.

T4.0 Does the proposed study qualify for 'exempt' IRB review or for a determination of either 'not research' or 'no human subject' involvement?

*no

T5.0 Does the proposed research study qualify for 'expedited' IRB review status?

*yes

Section: Cover Sheet

[reviewer notes¬]

Cover Sheet Section

CS1.0 What is the reason for this submission?

New Research Protocol Submission

CS1.1 Has this research study been approved previously by the University of Pittsburgh IRB?

* no

If the study expired or if this is a paper conversion, you are required to upload the last approved protocol and consent document, a completed Research Study Renewal Report Form found on the IRB website and a Data and Safety Monitoring Report.

Upload the last approved protocol, consent document, Renewal Report Form and Data and Safety Monitoring Report:

Name Modified Date

Previous IRB #:

CS1.1.1 Has this research study (or a substantially similar research study) been previously disapproved by the University of Pittsburgh IRB or, to your knowledge, by any other IRB?

* no

If Yes, identify the IRB, IRB number if Pitt IRB disapproved, and the primary reasons for disapproval:

[reviewer notes¬]

Cover Sheet Section

CS2.0 Title of Research Study:

Texting to Reduce Alcohol Consumption (TRAC) Trial

CS2.0.1

Requested approval letter wording:

Consent form

CS2.1 Research Protocol Abstract:

Alcohol consumption, especially in the form of heavy episodic drinking (bingeing), is common among young adults (Wechler, 2009). Despite high rates of illness and injury associated with heavy episodic drinking, many young adults are not aware of the risks (Edlund, 2009), few seek help for their drinking (SAMHSA, 2010) and many at-risk are not exposed to prevention-based interventions (Calabria BV, 2011). Opportunistic screening in hospital Emergency Departments (EDs) tied to behavioral interventions has the potential to prevent future alcohol-related harm among young adults (Monti, 2010; Mello, 2008), but efficacy across outcomes has been mixed (Havard, 2009) and large-scale implementation of prevention programs is low (Cunningham, 2010). Additionally, considerable gaps exist regarding predictors of drinking behavior and related consequences associated with drinking episodes among young adults exposed to interventions.

Prior research has documented that negative alcohol-related events can promote changes to drinking behavior, motivations for change, and cognitions related to drinking (Sobell). Ecological momentary assessment (EMA) is a sampling technique well suited to elucidate the event-level perspective to examine the interrelationships of expectancies, drinking, and consequences (Shiffman, 2009). It allows for real-time assessment of behavior and beliefs/feelings in the patient’s natural environment, improving validity of findings. Given the rapidly growing use of cell phone text-messaging (SMS) as a primary form of communication among young adults (Neilson, 2010), EMA performed through SMS allows for increased fidelity to assessments and patient participation. By using reports from pre-weekend expectancies and post-weekend drinking and consequences, we can ensure temporal relationships, minimize recall bias, investigate reciprocal relationships and examine both within- and between-person relationships. Findings from our own study support the use of SMS to collect alcohol use and motivation-related information from young adults (Suffoletto, 2012), but the associations between expectancies, drinking and consequences remains unexplored. We are currently recruiting young adults identified in the ED with hazardous drinking behavior in a 3-arm randomized controlled trial to test the hypothesis that exposure to a 12-week SMS program will result in immediate (3-month) and lasting (6-, and 9-month) decreases in alcohol consumption.

[reviewer notes¬]

Cover Sheet Section

CS3.0 Name of the Principal Investigator:

Brian Suffoletto

Note: Adjunct faculty of the University, including lecturers and instructors, are not permitted to serve as a PI or Faculty Mentor but may serve as co-investigators.

CS3.1 Affiliation of Principal Investigator:

UPitt faculty member

If your answer was Other, fill in the Principal Investigator's affiliation:

If you chose any of the Pitt options, please indicate the specific campus:

Main Campus - Pittsburgh

If you chose the UPitt faculty member option, provide the PI’s University Faculty Title:

Assistant Professor

CS3.1.1 Indicate below the name of the qualified University faculty member or UPP or UPMC staff member who will serve as a mentor and provide supervision or guidance regarding the conduct of this research study.

CS3.2 Address of Principal Investigator:

Iroquois Building, Suite 400A3600 Forbes Avenue Pittsburgh, PA 15261

CS3.3 Recorded Primary Affiliation of the Principal Investigator:

U of Pgh | School of Medicine | Emergency Medicine

CS3.4 Identify the School, Department, Division or Center which is responsible for oversight of this research study:

U of Pgh | School of Medicine | Emergency Medicine

CS3.5 Telephone Number of Principal Investigator:

412-901-6892

CS3.6 Recorded Current E-mail Address of Principal Investigator to which all notifications will be sent:

suffbp@upmc.edu

CS3.7 Fax Number:

412-547-6999

CS3.8 Does this study include any personnel from Carnegie Mellon University, and/or use any CMU resources or facilities (e.g., Scientific Imaging and Brain Research Center (SIBR)?

* no

CS3.9 Is this your first submission, as PI, to the Pitt IRB?

* no

[reviewer notes¬]

Cover Sheet Section

CS4.0 List of Co-Investigators:

Last First Organization

Abboud Andrew Other

Ahmed Naadia U of Pgh | Financial Aid | General University Budget Only

Amin Paula U of Pgh | School of Medicine | Critical Care Medicine

Begun Ryan U of Pgh | School of Engineering | Bioengineering Program

Bhat Nisha U of Pgh | Faculty of Arts and Sciences | Neuroscience

Bhatte Neeti U of Pgh | School of Medicine | Pathology

Bianco Victoria U of Pgh | Faculty of Arts and Sciences | Biological Sciences

Buhay Megan U of Pgh | Faculty of Arts and Sciences | Neuroscience

Clark Duncan U of Pgh | School of Medicine | Psychiatry

Coppler Patrick U of Pgh | Associate Vice Chancellor of Human Resources | All Temps

Delia Melissa Other

Ferruzza Kathryn U of Pgh | School of Medicine | Critical Care Medicine

Fraser Danyelle U of Pgh | School of Medicine | Surgery | T.E. Starzl Transplant Institute

Gaim Helen U of Pgh | School of Medicine | Critical Care Medicine

Gilchrist Erin U of Pgh | School of Medicine | Critical Care Medicine

Grady Kylie U of Pgh | School of Medicine | Critical Care Medicine

Hawley Alec U of Pgh | Faculty of Arts and Sciences | Psychology

Hellman Rachel UPMC | Other

Huerbin Sydney U of Pgh

Killeen Brian U of Pgh | School of Medicine | Critical Care Medicine

Koehler Janara Other

Korpon Jonathan U of Pgh | Faculty of Arts and Sciences | Neuroscience

Kristan Jeffrey U of Pgh | School of Medicine | Emergency Medicine

McManigle Kyle U of Pgh | School of Medicine | Emergency Medicine

Mealy Shane U of Pgh | School of Medicine | Critical Care Medicine

Moorhead Jeffrey U of Pgh | School of Health and Rehabilitation Sciences | Physical Therapy

Moyer Mitchell U of Pgh | School of Medicine | Critical Care Medicine

Ogunmola Ayodele Other

Person Mecca Laurel U of Pgh | University Center for Social and Urban Research | University Center for Social and Urban Research

Reinhart Evan U of Pgh | School of Health and Rehabilitation Sciences | Emergency Medicine

Russell Margaret U of Pgh | School of Medicine | Critical Care Medicine

Siegel Jordan U of Pgh | School of Medicine | Critical Care Medicine

Truong Sandra U of Pgh | Associate Vice Chancellor of Human Resources | All Temps

[reviewer notes¬]

Cover Sheet Section

CS5.0 Name of Primary Research Coordinator:

Jeffrey Kristan

CS5.1 Address of Primary Research Coordinator:

Iroquois Building Suite 400A

Pittsburgh PA 15261

CS5.2 Telephone Number of Primary Research Coordinator:

412-864-1919

CS6.0 Name of Secondary Research Coordinator:

CS6.1 Address of Secondary Research Coordinator:

CS6.2 Telephone Number of Secondary Research Coordinator:

CS6.3 Key Personnel/Support Staff (Only list those individuals who require access to OSIRIS):

Last First Organization

Morgan Maureen U of Pgh | School of Medicine | Emergency Medicine

[reviewer notes¬]

Cover Sheet Section

CS7.0 Will this research study use any Pediatric PittNet or Clinical and Translational Research Center (CTRC) resources?

no

CS7.1 Please select the sites you intend to use:

There are no items to display

[reviewer notes¬]

Cover Sheet Section

CS8.0 Select the entity responsible for scientific review.

Department Review - (a dean, department chair, division chief, or center head)

Note: DoD funded studies require departmental review

CS8.1 Select the school, department or division which is responsible for scientific review of this submission.

U of Pgh | School of Medicine | Emergency Medicine

CS8.1 Select the CTRC which is responsible for scientific review of this submission

[reviewer notes¬]

Cover Sheet Section

CS9.0 Does this research study involve the administration of an investigational drug or an FDA-approved drug that will be used for research purposes?

*no

CS9.1 Do you plan to utilize the Investigational Drug Service (IDS) to dispense the drug?

*

CS10.0 Is this research study being conducted under a University of Pittsburgh-based, sponsor-investigator IND or IDE application?

* no

If YES, you are required to submit the IND or IDE application and all subsequent FDA correspondence through the Office for Investigator-Sponsored IND and IDE Support (O3IS). Refer to applicable University policies posted on the O3IS website (www.o3is.pitt.edu).

CS10.1 Append to this application:

(1) Copy of the current version of the clinical protocol submitted with the IND or IDE application which corresponds to this IRB submission:

Name Modified Date

(2) Copy of the FDA’s letter which acknowledges receipt of the application and assignment of the IND or IDE number:

Name Modified Date

[reviewer notes¬]

Cover Sheet Section

CS11.0 Use the 'Add' button to upload one or more of the following:

the sponsor protocol (including investigator initiated studies) and/or other brochures

the multi-center protocol and consent form template, if applicable

Name Modified Date

Is this research study supported in whole or in part by industry? This includes the provision of products (drugs or devices).

* no

Is this a multi-centered study?

* no

[reviewer notes¬]

Cover Sheet Section

CS12.0 Does your research protocol involve the evaluation or use of procedures that emit ionizing radiation?

* no

HUSC GUIDANCE

REQUIREMENTS FOR THE REVIEW OF HUMAN SUBJECT RESEARCH PROTOCOLS BY THE

HUMAN USE SUBCOMMITTEE (HUSC), RADIATION SAFETY COMMITTEE

For Research Protocols Involving the Evaluation of Use of Diagnostic Procedures that Emit Ionizing Radiation:

Formal HUSC review/approval is required if the research protocol involves any of the following:

The use or evaluation of a radioactive agent or procedure that is not currently approved (i.e., for any clinical indication) by the FDA

The evaluation (i.e., for safety and/or effectiveness) of a FDA-approved radiopharmaceutical or procedure for an “off label” indication1; or the use of a FDA-approved radiopharmaceutical or procedure for an “off label” indication if such use is experimental (i.e., not routinely performed in clinical practice).

Individuals (e.g., healthy volunteers) who would not be undergoing the procedure in association with the diagnosis or treatment of a disease or condition

Formal HUSC review/approval is not required if the diagnostic procedure is being performed, in a standard clinical manner and frequency, for screening or to evaluate the outcome of a treatment regimen. This would include diagnostic procedures for off- label uses that are routinely performed in clinical practice. 2,3

For Research Studies Involving the Use or Evaluation of Therapeutic Procedures that Emit Ionizing Radiation:

Formal HUSC review/approval is required if parameters (e.g., total radiation dose, dose fractionation scheme, etc.) of the radiation therapy procedure(s) are defined by the research protocol.

1An “off-label” indication is a clinical indication which is not currently specified in the FDA-approved product labeling.

2The risks of radiation exposure associated with the diagnostic procedure must continue to be addressed in the protocol and consent form using the HUSC-accepted wording.

3The University of Pittsburgh IRB, at its discretion, may request formal HUSC review of the research protocol.

For any questions related to these requirements or their application, contact the Chair of the HUSC (412-383-1399) or the University’s Radiation Safety Office (412-624-2728)

CS12.1 After reviewing the HUSC guidance above, does your research protocol require HUSC review? (Note: University of Pittsburgh’s Radiation Safety Committee oversight is limited UPMC Presbyterian-Shadyside, Magee Women’s Hospital of UPMC, Children’s Hospital of Pittsburgh-UPMC, and Hillman Cancer Center. If other sites, you will be required to obtain approval from your radiation safety officer. Please contact askirb@pitt.edu for more information.)

Upload Radiation Forms:

Name Modified Date

CS13.0 Does this research study involve the deliberate transfer of recombinant DNA (rDNA) or DNA or RNA derived from rDNA into human subjects?

* no

Upload Appendix M of NIH Guidelines:

Name Modified Date

CS14.0 Are you using UPMC facilities and/or UPMC patients during the conduct of your research study?

* no

If Yes, upload completed Research Fiscal Review Form:

Name Modified Date

[reviewer notes¬]

Cover Sheet Section

CS15.0 Indicate the sites where research activities will be performed and/or private information will be obtained.

Choose all sites that apply and/or use Other to include sites not listed:

Sites:

UPMC

University of Pittsburgh

Campus:

There are no items to display

List university owned off-campus research sites if applicable:

UPMC

Sites:

UPMC Presbyterian

UPMC Magee Women's Hospital

UPMC Mercy

UPMC Shadyside

UPMC Cancer Network Sites:

Site

There are no items to display

If you selected School, International or Other, list the sites:

*For non Pitt or UPMC entities, upload documents granting permission to conduct research at that site:

Name Modified Date

CS15.1 Have you, Brian Suffoletto , verified that all members of the research team have the appropriate expertise, credentials, and if applicable, hospital privileges to perform those research procedures that are their responsibility as outlined in the IRB protocol?

* yes

CS15.2 Describe the availability of resources and the adequacy of the facilities to conduct this study:

* Together, UPMC Mercy, Presbyterian, Shadyside and Magee sees over 200,000 adult patients per year. Each site has private care rooms to screen, enroll and interview patient-participants.

[reviewer notes¬]

Cover Sheet Section

CS16.0 Special Research Subject Populations:

Categories

None

[reviewer notes¬]

Cover Sheet Section

CS17.0 Does your research involve the experimental use of any type of human stem cell?

* no

Section: Section 1 - Objective, Aims, Background and Significance

[reviewer notes¬]

Section 1 - Study Objective, Specific Aims, Background and Significance

1.1 Objective: What is the overall purpose of this research study? (Limit response to 1-2 sentences.)

This project will confirm whether or not we can initiate and maintain reduced alcohol consumption among at-risk young adults using a text-message program.

1.2 Specific Aims: List the goals of the proposed study (e.g., describe the relevant hypotheses or the specific problems or issues that will be addressed by the study).

Primary Aim: Determine whether SMS-based intervention reduces the number of binge drinking episodes, drinks per drinking occasion, alcohol screening status and adverse consequences of alcohol use in hazardous drinking young adults identified in the ED. We hypothesize that at 3 months post-ED care, Arm 1 (EA+I) will report greater reductions in alcohol consumption than Arms 2 (EA) and 3 (Control). Furthermore, we hypothesize that assessment reactivity will result in some reductions in alcohol consumption in Arm 2 (EA) relative to Arm 3 (Control). We will explore the lasting effect of EA+I on alcohol consumption outcomes compared to EA and control at 6 and 9 months.

Secondary Aims: Secondary aims will examine the relationships between alcohol expectancies and alcohol use; (2) study bidirectional influences of expectancies, consequences, and drinking over time; (3) examine whether the immediate consequences of drinking change subsequent expectancies and drinking over time; and (4) examine the time-varying and time-constant moderators of the within-person relationships between expectancies, drinking, and consequences.

1.3 Background: Briefly describe previous findings or observations that provide the background leading to this proposal.

In a pilot study (PART), we were able to assess the feasibility of screening young adults in the ED for hazardous drinking and recruiting them for a SMS-based interventional trial, collect weekly drinking data through SMS and describe the variance in drinking outcomes. Young adults in our three urban EDs (n=45; aged 18-24 years, 54% female) identified as hazardous drinkers by the Alcohol Use Disorders Identification Test-Consumption (AUDIT-C) score were randomly assigned to weekly SMS feedback with goal setting (EA+I), weekly SMS drinking assessments without feedback (EA) or Control. We screened 109 young adults across 24 unique days and 52 (48%; 95% CI 38-50) screened-positive for hazardous drinking. Of these, 45 (87%; 95% CI 74-94) met inclusion criteria, were enrolled and randomized, and 6 (13%; 95% CI 5-27) did not complete 3-month web-based follow-up. 88% (95% CI 84-91%) of weekly SMS drinking assessments were answered, with 77% (95% CI 58-90) of participants responding to all 12 weeks. 65% of participants replied to SMS drinking queries within 1 minute of being sent. Additionally, patients reported that their SMS-based drinking assessments were highly accurate (median 7/7, IQR 6-7) and felt very comfortable (median 7/7; IQR 6-7) sharing their drinking habits via SMS. Agreeing to set a goal via SMS was associated with a repeat binge episode 36% (95% CI 17-55) of the time compared to 63% (95% CI 44-81) when not willing to set a goal. At 3-months, EA+I participants had 3.4 (SD 5.4) fewer binge episodes in the last month and 2.1 (SD 1.5) fewer drinks per drinking day when compared to baseline.

1.4 Significance: Why is it important that this research be conducted? What gaps in existing information or knowledge is this research intended to fill?

Young adults may be at the greatest risk for alcohol-related harm. Access to and use of treatment services for alcohol use disorders in young adults is low. The Emergency Department is an opportunistic setting to intervene to reduce future harm, but alcohol programs are underutilized. This proposal will make a valuable contribution to advancing the understanding the processes of changes in drinking behavior and event-level consequences associated with drinking episodes among young adults.

Section: Section 2 - Research Design and Methods

[reviewer notes¬]

Section 2 - Research and Design Methods

2.1 Does this research study involve the use or evaluation of a drug, biological, or nutritional (e.g., herbal or dietary) supplement?

* no

2.1.1 Does this research study involve an evaluation of the safety and/or effectiveness of one or more marketed nutritional (e.g., herbal or dietary) supplements for the diagnosis, prevention, mitigation or treatment of a specific disease or condition or symptoms characteristic of a specfic disease or condition?

*

2.1.1.1 List each of the marketed nutritional supplements being evaulated in this research study. Specify for each supplement the corresponding IND number or attach FDA correspondence specifying that an IND is not required.

Marketed nutritional supplement IND number

There are no items to display

Upload FDA correspondence specifying that an IND is not required, if applicable:

Name Modified Date Version

[reviewer notes¬]

Section 2 - Research Design and Methods

2.2 Will this research use or evaluate the safety and/or effectiveness of one or more devices?

* no

2.2.1 Does this research study involve an evaluation of the safety and/or effectiveness of one or more devices not currently approved by the FDA for general marketing?

*

If YES, describe your plan to prevent unauthorized use of the investigational device:

2.2.1.1 List each of the unapproved devices being evaluated in this research study.

Specify for each listed device the corresponding Investigational Device Exemption (IDE) number or provide a justification for why you feel that this device and its use, as proposed in this research study constitute a non-significant risk (i.e., to include potential failure of the device) to the research subjects:

Unapproved device IDE # Non-significant risk justification

There are no items to display

[reviewer notes¬]

Section 2 - Research Design and Methods

2.3 Summarize the general classification (e.g., descriptive, experimental) and methodological design (e.g., observational, cross-sectional, longitudinal, randomized, open-label single-blind, double-blind, placebo-controlled, active treatment controlled, parallel arm, cross-over arm) of the proposed research study, as applicable.

Experimental, longitudinal, randomized, active treatment controlled.

2.3.1 Does this research study involve a placebo-controlled arm?

* no

[reviewer notes¬]

Section 2 - Research Design and Methods

2.4 Will any research subjects be withdrawn from known effective therapy for the purpose of participating in this research study?

* no

2.4.1 Provide a justification for discontinuing subjects from known effective therapy for the purpose of study participation.

2.4.2 Describe the risks to subjects associated with discontinuing them from known effective therapy for the purpose of study participation.

[reviewer notes¬]

Section 2 - Research Design and Methods

2.5 Will screening procedures (i.e., procedures to determine research subject eligibility) be performed specifically for the purpose of this research study?

* yes

2.5.1 List the screening procedures that will be performed for the purpose of this research study. Do NOT include the inclusion/exclusion criteria in this section as they will be addressed in section 3; questions 3.13 and 3.14.

An investigator will conduct confidential screening of ED patients during breaks in patient medical care. All responses to screening questions will be recorded on a secure web site. An investigator will identify male and female patients between ages 18 and 25 years who receive medical care in the ED through the Triage Report (name, age, chief complaint, and ED bed #) viewed from the ED tracking software. This type of screening is routine in the ED, and can be done without accessing the patient chart or identifiers. The investigator will then ask the clinician (physicians, nurses or physician extenders) caring for a particular patient to ask if that patient would be interested in hearing about the study. ED providers are instructed to refer only patients who are able to provide informed consent (i.e., oriented, able to concentrate, and can understand/remember requirements of the study) and to document in their medical record about the patient's assent to talk with an investigator.

An investigator will approach all permission-granting, potentially eligible patients to obtain informed consent for study screening. To ensure privacy, family or other individuals accompanying the patient will be asked to leave the area prior to the screening procedures and patients will be reminded that other people may be able to overhear the conversation. The investigator will provide tablet computer for participants to complete screening questionnaire immediately after informed consent.

Screening will include completion of 13 questions taken from the CDC's Youth Risk Behavior Survey (YRBS), 4 questions to determine alcohol use (AUDIT-C) and 3 additional questions to determine eligibility. We believe that this screening is necessary to determine how those who screen and/or enroll in our study differ from the general ED population of young adults. Screened participants who have a total score of >3 for women or>4 for men on the 3 AUDIT-C questions AND report having any drinking episode over the prior 30 days with >3 drinks will meet inclusion criteria. We will exclude those who do not currently have a cell phone with SMS, those who have been diagnosed with an alcohol or substance use disorder, and those with current treatment for a psychiatric disorder.

Upon completion of screening questions, the participant returns the tablet computer to an investigator, who then enters a unique pass code. The computer shows the investigator whether the participant is eligible or not, and whether the AUDIT-C is positive or not. The RA will then read a standard script based on eligibility and AUDIT-C status report. Screening for and addressing hazardous drinking is not currently part of usual care for ED patients at any of the three sites. Therefore, young adults with hazardous drinking who are not eligible or decline study participation will be provided the same referral and educational materials as study participants, but they will not participate in any further assessments or monitoring. These young adults will be directed to contact their primary care physician or a local community resource about reducing their alcohol consumption.

2.5.2 What steps will be taken in the event that a clinically significant, unexpected disease or condition is identified during the conduct of the screening procedures?

Not Applicable

[reviewer notes¬]

Section 2 - Research Design and Methods

2.6 Provide a detailed description of all research activities (e.g., all drugs or devices; psychosocial interventions or measures) that will be performed for the purpose of this research study.

This description of activities should be complete and of sufficient detail to permit an assessment of associated risks.

At a minimum the description should include:

all research activities

personnel (by role) performing the procedures

location of procedures

duration of procedures

timeline of study procedures

An investigator will offer eligible patients study participation and obtain written informed consent. The RA will present an oral and written description of the study as part of informed consent, including a description of the project procedures, potential risks and benefits, and confidentiality. Patients will be informed that if they agree to participate, they will receive up to a $100 payment. It will be made clear that participation may include up to receipt of 120 SMS over 12 weeks, about half of which require brief SMS responses. All participants will be advised to set up password protection on their cell phones and to erase messages after responding to minimize the chance of loss of private information. Patients will be informed that participation will include three paid follow-up web assessments.

An investigator will provide tablet computer for participants to complete baseline questionnaires immediately prior to randomization. Participants in all Arms complete 13 questions covering demographics, drug use, and a 30-day Timeline Follow Back (TLFB) calendar about alcohol use (Sobell & Sobell, 1992).

All participants will be asked to provide two friends or family members who will be contacted in the event that the research staff is unable to reach the patient. Information for these “locators” will include name, address, phone numbers, relation to the patient, and the best times of day to reach the locator. Participants will be asked to sign a form letter addressed to their locator that explains that they are participating in a research project and that they have named this person as a locator. Participants will be compensated $10.00 for completion of baseline instruments.

Randomization assignments will be generated in blocks of 8 for each site by the study statistician and allocated electronically. Randomization sequences will be stratified by ED presentation attributable to alcohol in a 2 EA+I: 1 EA: 1 Control ratio to allow for more observations to be available for analyses of mediators and moderators of positive outcomes in the EA+I group.

The EA+I intervention is adapted from our previous study (Suffoletto, 2012), and incorporates features directly addressing cognitive determinants of binge drinking. All SMS are less than 160 characters and read at no more than a 6th grade level. SMS consists of (1) pre-weekend drinking intention assessments followed by a motivational dialogue and negotiation of an individual goal for the coming weekend and (2) post-weekend drinking and injury assessments with feedback. A computer selects the dialogue from a library and delivers the dialogue to the participant’s cell phone through SMS. The EA group will receive identical post-weekend alcohol assessments to the EA+I condition. After each assessment, the computer will send an SMS statement: “Your response has been received and is appreciated.” To ensure parallel attention and number of contacts across groups, Control group participants will receive weekly SMS for 12 weeks to remind them to follow-up for 3-month web-based assessments, “Look for our text in [X] weeks to complete your final survey,” where [X] was the number of weeks until study completion.

2.6.1 Will blood samples be obtained as part of this research study?

* no

If Yes, address the frequency, volume per withdrawal, the total volume per visit, and the qualifications of the individual performing the procedure:

Study Flow Chart:

Name Modified Date

[reviewer notes¬]

Section 2 - Research Design and Methods

2.7 Will follow-up procedures be performed specifically for research purposes? Follow-up procedures may include phone calls, interviews, biomedical tests or other monitoring procedures.

* yes

Detailed procedures listed in the textbox below:

Follow-up will be conducted over a password-protected web site at 3, 6, and 9 months. For those participants who do not have access to a computer to complete follow-up, a research staff member, blinded to treatment assignment, will administer the questionnaire over the phone. We expect each follow-up assessment to take approximately ten minutes. To improve subject retention, we will email and text-message subjects one day prior to each of their follow-ups. We will ask participants to email us if they are planning on changing phone numbers in the next few months. For those participants who do not complete follow-up by 5 days following intended completion, we will email and/or call them. If we are unable to reach them, we will contact their “locator” to inquire about participant contact information. Participants will be compensated $20.00 for 3 month web-based assessment, $30.00 for 6-month and $40.00 for completion of 9-month web-based assessments. All payments will be made using Pitt Debit cards. Screening and baseline assessments will be collected on a web based survey system (WebDataXpress) using a touchscreen tablet computer using the hospital’s WiFi network. Patient responses are encrypted prior to transmission to and from the server that hosts the software, meeting the standards of Human Subjects Protection.

We will also invite participants randomized to the intervention who have completed the 9-month follow-up to participate in focus groups to discuss their experience with the TRAC program. We will email all eligible candidates and invite them to participate using a standardized script (attached). Interested subjects will be asked to contact the study coordinator by telephone to arrange a scheduled time to review the informed consent and ask questions. During this call, the coordinator will explain the study components, procedures, and the inclusion/exclusion criteria and answer questions related to the study. The coordinator will obtain the person's name and contact information for scheduling. We will conduct 4 focus groups with no more than 5 participants per group, and use an elicitation guide (attached). For those not able to come in for a focus group, we will ask them if they could complete a brief phone interview.

[reviewer notes¬]

Section 2 - Research Design and Methods

2.8 Does this research study involve the use of any questionnaires or survey instruments?

* yes

Upload a copy of all unpublished surveys/questionnaires. Also upload any published materials that may include questions, images, video, or sound recordings that may be especially disturbing to subjects:

Name Modified Date

Focus Group Survey 4/21/2014 5:42 PM

TRAC_codebook.xlsx 8/20/2012 11:07 AM

List the name and publisher for commercially available materials (Note: these materials do not need to be uploaded):

[reviewer notes¬]

Section 2 - Research Design and Methods

2.9 If subjects are also patients, will any clinical procedures that are being used for their conventional medical care also be used for research purposes?

* no

If Yes, describe the clinical procedures (and, if applicable, their frequency) that will be used for research purposes:

2.10 The blood sample question was moved to 2.6.1.

[reviewer notes¬]

Section 2 - Research Design and Methods

2.11 What is the total duration of the subject's participation in this research study across all visits, including follow-up surveillance?

* 12 months

[reviewer notes¬]

Section 2 - Research Design and Methods

2.12 Does this research study involve any type of planned deception?

If Yes, you are required to request an alteration of the informed consent process (question 4.7)

* yes

2.12.1 Describe the planned deception:

*

We will not overtly describe the arms of the study in the informed consent process.

2.12.2 Provide a justification for this planned deception:

*

We found in our pilot study that intervention participants may change their drinking behavior after enrollment but prior to intervention exposure (Week 1), suggesting that the expectancy of being in the SMS intervention group could have an effect on their behavior. Therefore, we wish to withhold their knowledge of which arm they were randomized to. Instead, we will tell them that they can expect to receive up to 220 SMS over the next 12 weeks, but as few as 12. We will ask them to respond as honestly as possible to all questions, if they receive them.

2.12.3 Describe when and how subjects will be debriefed:

*

Their involvement will become self-evident by the end of the intervention.

[reviewer notes¬]

Section 2 - Research Design and Methods

2.13 Does this research study involve the use of UPMC/Pitt protected health information that will be de-identified by an IRB approved "honest broker" system?

* no

2.13.1 Identify the name of the honest broker system:

2.13.2 Specify the IRB-assigned honest broker system number (e.g., HB123456):

2.13.3 Specify the names of the individuals who will provide the honest broker services:

2.13.4 Upload the signed honest broker assurance agreement:

Name Modified Date

There are no items to display

[reviewer notes¬]

Section 2 - Research Design and Methods

2.14 Will protected health information from a UPMC/Pitt HIPAA covered entity be accessed for research purposes or will research data be placed in the UPMC/Pitt medical record?

* no

If you answer Yes, you are required to submit this study to the Center for Assistance in Research using e-Record (CARe). Per UPMC Policy HS-RS0005, all research projects that access or involve UPMC electronic protected health information (e-PHI) must be submitted to CARe, with the exception of clinical trials that are contracted through the UPMC Office of Sponsored Programs and Research Support (OSPARS).

Complete the online submission form at http://www.eresearch.pitt.edu/request.aspx. After the study is submitted in OSIRIS, a CARe representative will conduct a review. You will be notified once your CARe review is complete or if anything further is needed.

Studies that will access only paper-based medical records (not in combination with any electronic records) do not need to be submitted to CARe.

For additional information, please see https://care.upmc.com.

Describe the medical record information that will be collected from the UPMC/Pitt HIPAA covered entity and/or the research-derived information that will be placed in the medical records.

2.14.1 Will protected health information from a non-UPMC/Pitt HIPAA covered entity be obtained for research purposes or will research data be placed in the non-UPMC/Pitt medical record?

* no

If Yes, describe how the HIPAA requirements will be met:

I, Brian Suffoletto, certify that any member of my research team accessing, reviewing and/or recording information from medical records have completed HIPAA Researchers Privacy Requirements (Formerly RPF Module 6) training. The HIPAA certificates must be available for review if audited but do not need to be uploaded into this OSIRIS application.

*

2.14.2 Are you requesting a waiver of the requirement to obtain written HIPAA authorization for the collection of the PHI from a UPMC/Pitt covered entity? Note that the University of Pittsburgh IRB cannot grant a HIPAA waiver for entities outside of UPMC/Pitt.

*

[reviewer notes¬]

Section 2 - Research Design and Methods

2.15 Does this research study involve the long-term storage (banking) of biological specimens?

* no

2.15.1 Broadly describe the intended future use of the banked biological specimens:

2.15.2 Indicate the planned length of storage of the banked biological specimens:

*

2.15.3 Will biological specimens be stored without identifiers or linkage codes?

*

[reviewer notes¬]

Section 2 - Research Design and Methods

2.16 Will research participants be asked to provide information about their family members or acquaintances?

* yes

2.16.1 Describe what information about the third party will be obtained from the participant:

We will ask participants to provide the first names, phone numbers and relationship of at least 2 people. We will use these people to find the participants if we lose contact.

2.16.2 If the information about the third party is of a private nature, can the identity of the third party be readily ascertained or associated with this information?

*

No

Describe the private information that will be collected and recorded about the third party:

We will ask only for first name, phone number and relationship to the participant. We will only ask these people to provide updated contact information for the participant if we lose ocntact. We will not disclose the nature of the participant involvment in research.

[reviewer notes¬]

Section 2 - Research Design and Methods

2.17 What are the main outcome variables that will be evaluated in this study?

Self-report of prior 30 day alcohol consumption at 3-months, 6-months, and 9-months.

2.18 Describe the statistical approaches that will be used to analyze the study data.

* Addressed below:

We will test the effects of EA+I, compared to EA and Control, on alcohol use variables (binge drinking episodes, drinks per drinking occasion) at 3,6,and 9 months. These analyses use data from the 30-day TLFB completed at each follow-up. Depending on the nature of the variable, the analyses will use either a normal distribution or negative binomial model for count data (e.g., number of binge drinking days). Analyses will control for age, gender, and the relevant covariates as assessed at baseline. A linear effect of time also will be included. The independent variable of main interest will be intervention type, which will be coded using group indicator variables. Our models will account for clustering within each subject and each site. If any covariates are identified as necessary during preliminary analyses, these will also be included as covariates. After testing the main effects of condition, we will test interactions between intervention and time to inform us whether differences in alcohol use and consequences outcomes associated with intervention become less or more pronounced over 12 months. Following “intention-to-treat” principles, analyses will be conducted on all who were randomized to a condition regardless of whether they actually completed the intervention sessions.

[reviewer notes¬]

Section 2 - Research Design and Methods

2.19 Will this research be conducted in (a) a foreign country and/or (b) at a site (e.g., Navajo Nation) where the cultural background of the subject population differs substantially from that of Pittsburgh and its surrounding communities?

* no

Note that copies of training records, licenses, certificates should be maintained in the study regulatory binder and are subject to audit by the Research Conduct and Compliance Office (RCCO).

In addition, individuals planning to conduct human subject research outside the United States must complete an optional module on the CITI training website: International Studies. Click here to access the instruction sheet for accessing optional CITI modules.

2.19.1 Address the following for each of the foreign/culturally different sites where this research will be conducted:

• Name of site

• Name of authorized individual (e.g., IRB Chair) from the local IRB or other human subject protections entity that is responsible for the review and approval of the project; upload approval letter with an English translation, if applicable

• Name and qualifications of the site collaborator responsible for the conduct of the research (e.g., site PI)

• The anticipated number of subjects that will be enrolled at that site

• If Federally funded, provide the Federalwide Assurance number (FWA) assigned to the site

*

Site Date Modified

There are no items to display

2.19.1.1 Provide a description of the context of cultural norms and local laws and highlight differences between U.S. culture in all areas relevant to your study, including, at a minimum:

• Age of majority of participants to be enrolled

• If study includes minors or decisionally impaired subjects, summarize laws on guardianship

• If your study involves any invasive medical procedure (including blood draws), provide assurance that the individuals undertaking those procedures for research purposes are appropriately credentialed.

• If your study involves the administration of a drug, device or biologic for research purposes, describe the process for shipping, labeling, storing and dispensing, and indicate how these are consistent with all relevant local (and US) laws, including those requiring import / export permits.

• If your study involves collection of biological specimens, describe the process for shipping, labeling, storing and using such samples. Identify any special local consent requirements, and any special permits that may be required by local law.

*

2.19.1.2 Describe any aspects of the local cultural, political or economic climate that might increase the risks of harm for either local participants or researchers. Describe the steps you will take to minimize these risks. Review Pitt Guidelines and Travel Warnings and Alerts for important information.

*

2.19.2 Will all individuals being recruited to participate in this research study be able to read and comprehend English

*

If No, describe how consent will be obtained. Explain provisions for culturally appropriate recruitment and consent accommodations such as, translations or involvement of native language speakers, especially if literacy is not widespread in this country.

2.19.2.1 If translated documents are used, upload a letter from the translator certifying the accuracy of the translation:

(Translator Certification Form is available under the Resources tab located to right of this item)

Name Modified Date

There are no items to display

2.19.3 Will all of the research procedures described in this IRB application be conducted at the foreign/culturally different sites?

* Yes No

If No, describe the subset of research procedures to be performed at the sites:

2.19.4 To what extent do the local site requirements to protect subject confidentiality and privacy differ from US standards. If applicable, explain how those will be addressed by this research team:

*

2.19.5 If the researcher is a student, describe how the student will communicate with the advisor during the conduct of the research and how the advisor will oversee the research

[reviewer notes¬]

Section 2 - Research Design and Methods

2.21 Will this research study be conducted within a nursing home located in Pennsylvania?

* no

2.21.1 Does this research involve a medical procedure or an experimental treatment?

*

2.21.2 Does the research study involve the exposure of nursing home residents to pain, injury, invasion of privacy, or ask the resident to surrender autonomy?

*

If Yes to either question, upload the Pennsylvania Department of Health approval letter:

Name Modified Date

Section: Section 3 - Human Subjects

[reviewer notes¬]

Section 3 - Human Subjects

3.1 What is the age range of the subject population?

18-25 years old

3.2 What is their gender?

* Both males and females

Provide a justification if single gender selected:

3.3 Will any racial or ethnic subgroups be explicitly excluded from participation?

* no

If Yes, identify subgroups and provide a justification:

3.4 For studies conducted in the U.S., do you expect that all subjects will be able to comprehend English?

* yes

If No, identify what languages will be understood by subjects:

3.4.1 If any documents have been translated, upload letter from the translator certifying the accuracy of the translation:

Name Modified Date

There are no items to display

[reviewer notes¬]

Section 3 - Human Subjects

3.5 Participation of Children: Will children less than 18 years of age be studied?

* no

If No, provide a justification for excluding children:

Drinknig among adolescents is different than in young adults.

3.5.1 Specify the age range of the children to be studied.

(Check all that apply below:)

*

Choices

There are no items to display

3.5.2 Provide a rationale for the specific age ranges of the children to be studied:

3.5.3 Describe the expertise of the study team for conducting research with children within this age range:

3.5.3.1 Have you obtained the following clearances from all research staff who may have direct contact with children under the age of 18? Direct contact under the law includes face-to-face, and telephonic or electronic, contact with minors. Please see the Child Clearances guidance document for further explanation?

Pennsylvania Department of Public Welfare Child Abuse History Clearance;

Pennsylvania State Police Criminal Record Check; and

FBI Criminal Background Check

Note: If No, once all clearances are obtained, a modification must be submittted.

If you selected N/A, please explain:

It is important to note that “direct contact” refers not only to face-to-face meetings but also extends to communication via phone (including text messaging), social media or internet. Direct contact also includes the care, guidance, supervision or control, or routine interaction with, minors. Conversely, a participating investigator or support staff member who does not have direct contact, either electronically or in person, with children does not need to obtain clearances (e.g., statistician, non-clinical laboratory personnel, etc.). If your research study provides babysitting services, the babysitters must have the required child clearances.

* Note: It is the responsibility of the principal investigator to ensure that all research staff have these clearances prior to any interaction with children. Contact Human Resources at 412-624-8150 for assistance with this process.

3.5.4 Describe the adequacy of the research facilities to accommodate children within this age range:*

3.5.5 Permitted Categories of Research: The Federal Policy and FDA regulations governing human subject protections specify that research involving children must fall into one of the following permitted categories.

*

45 CFR 46.406

The risk represents only a minor increase over minimal risk.

The research procedures present experiences to the subjects that are reasonably commensurate with those inherent in their actual or expected medical, dental, psychological, social, or educational situations.

The research procedures are likely to yield generalizable knowledge about the subjects’ disorder or condition which is of vital importance for understanding or amelioration of the subjects’ disorder or condition.

45 CFR 46.407

The risk is justified by the anticipated benefit to the subjects; and the relation of the anticipated benefit to the risk is at least as favorable to the subjects as that presented by available alternative approaches.

Provide a justification which must address all considerations related to the designated category of research:

[reviewer notes¬]

Section 3.0 - Human Subjects

3.6 Does this research study involve prisoners, or is it anticipated that the research study may involve prisoners?

* no

3.6.1 The Federal Policy and FDA regulations specify that research involving prisoners must fall into one of the following permitted categories.

*

*Provide a justification for your designation:

General Requirements: The Federal Policy and FDA regulations specify that research involving prisoners must also conform to each of the following general requirements. Describe how your study meets each of the following regulations.

3.6.2 Any possible advantages accruing to the prisoner through his or her participation in the research, when compared to the general living conditions, medical care, quality of food, amenities, and opportunity for earnings in the prison are not of such a magnitude that the prisoner’s ability to weigh the risks of the research against the value of such advantages in the limited choice environment of the prison is impaired. [45 CFR 46.305 (a)(2)]

*

3.6.3 The risks involved in the research are commensurate with risks that would be accepted by nonprisoner volunteers.

[45 CFR 46.305 (a)(3)]

*

3.6.4 The procedures for the selection of subjects within the prison are fair to all prisoners and immune from arbitrary intervention by prison authorities or prisoners.

[45 CFR 46.305 (a)(4)]

*

3.6.5 Information regarding the research is presented to the potential prisoners-subjects in a language which is understandable to them.[45 CFR 46.305 (a)(5)]

*

3.6.6 Adequate assurance exists that the parole boards will not take into account a prisoner’s participation in the research in making decisions regarding parole, and each prisoner is clearly informed in advance that participation in the research will have no effect on his or her parole. [45 CFR 46.305 (a)(6)]

*

3.6.7 Where there may be a need for follow-up examination or care of the prisoners-subjects after the end of their participation in the research, adequate provision has been made for such examination or care; taking into account the varying lengths of individual prisoners’ sentences, and the prisoners have been informed of this fact. [45 CFR 46.305 (a)(7)].

*

[reviewer notes¬]

Section 3 - Human Subjects

3.7 Will pregnant women be knowingly and purposely included in this research study?

* no

General Requirements: The Federal Policy [45 CFR 46, Subpart B] specify that research involving pregnant women and/or fetuses must also confirm to each of the following criteria. Describe how your study meets each of the requirements.

3.7.1 Where scientifically appropriate, preclinical studies, including studies on pregnant animals, and clinical studies, including studies on non-pregnant women, have been conducted and provide data for assessing potential risks to pregnant women and fetuses. [45 CFR 46.204 (a)] [Include references]

*

3.7.2 The risk to the fetus is caused solely by interventions or procedures that hold out the prospect of direct benefit for the women or the fetus; or, if there is no such prospect of direct benefit, the risk to the fetus is not greater than minimal and the purpose of the research is the development of important biomedical knowledge which cannot be obtained by any other means. [45 CFR 46.204 (b)]

*

3.7.3 Any risk is the least possible for achieving the objectives of the research. [45 CFR 46.204 (c)]

*

3.7.4 No inducements, monetary or otherwise, will be offered to terminate the pregnancy. [45 CFR 46.204 (h)]

*

3.7.5 Individuals engaged in the research will have no part in any decisions as to the timing, method, or procedures used to terminate a pregnancy. [45 CFR 46.204 (i)]

*

3.7.6 Individuals engaged in the research will have no part in determining the viability of a neonate. [45 CFR 46.204 (j)]

*

[reviewer notes¬]

Section 3 - Human Subjects

3.8 Does this research study involve neonates?

* no

General Requirements: The Federal regulations [45 CFR 46.205] specify that research involving neonates of uncertain viability and nonviable neonates must conform to each of the general requirements. Describe how each of the following requirements will be met.

3.8.1 Where scientifically appropriate, preclinical and clinical studies have been conducted and provide data for assessing potential risks to neonates (include references). [45 CFR 46.205 (a)(1)]

*

3.8.2 Individuals engaged in the research will have no part in determining the viability of the neonate. [45 CFR 46.205 (a)(3)]

*

3.8.3 Does this research study involve neonates of uncertain viability? [45 CFR 46.205(b)]

*

3.8.3.1 The Federal regulations specify that, until it is ascertained whether or not a neonate is viable, a neonate may not be involved in research unless one of the following conditions is met.

*

*Provide a justification for your selection:

3.8.4 Does this research study involve nonviable neonates? [45 CFR 46.205(c)]

*

General Requirements: The Federal regulations specify that, after delivery, a nonviable neonate may not be involved in research unless each of the following additional conditions are met [45 CFR 46.205(c)].

3.8.4.1 Vital functions of the neonate will not be artificially maintained. [45 CFR 46.205 (c)(1)]

*

3.8.4.2 The research will not terminate the heartbeat or respiration of the neonate. [45 CFR 46.205 (c)(2)]

*

3.8.4.3 There will be no added risks to the neonate resulting from the research. [45 CFR 46.205 (c)(3)]

*

3.8.4.4 The purpose of the research is the development of important biomedical knowledge that cannot be obtained by other means. [45 CFR 46.205 (c)(4)]

*

[reviewer notes¬]

Section 3 - Human Subjects

3.9 Fetal Tissues: Does this research involve the use of fetal tissues or organs?

* no

General Requirements: In accordance with the Pennsylvania Abortion Control Act, fetal tissues or organs may only be obtained for use in research subsequent to obtaining the written informed consent of the mother. The Pennsylvania Abortion Control Act specifies that research involving the use of fetal tissue or organs must also conform to each of the following requirements. [Indicate how you will conform to each requirement]

3.9.1 Informed consent for the research use of fetal tissue derived from an abortion will be obtained separate from, and after, the decision and consent to abort has been made.

*

3.9.2 No consideration of any kind (i.e., monetary or otherwise) will be offered to the mother in obtaining her consent for the research use of the fetal tissue or organs.

*

3.9.3 The mother will not be permitted to designate a recipient of the fetal tissue or organs for use in research.

*

3.9.4 All persons who participate in the procurement or use of the fetal tissue or organs will be informed as to the source of the tissue (e.g., abortion, miscarriage, stillbirth, ectopic pregnancy).

*

[reviewer notes¬]

Section 3.0 - Human Subjects

3.10 What is the total number of subjects to be studied at this site, including subjects to be screened for eligibility?

Note: The number below is calculated by summing the data entered in question 3.11. Any additions or changes to the values entered in 3.11 will be reflected in 3.10.

* 6000

3.11 Identify each of the disease or condition specific subgroups (include healthy volunteers, if applicable) that will be studied.

Click on the "Add" button and specify for each subgroup:

1) how many subjects will undergo research related procedures at this site; and

2) if applicable, how many subjects will be required to undergo screening procedures (e.g., blood work, EKG, x-rays, etc.) to establish eligibility. Do Not include subjects who will undergo preliminary telephone screening.

* Subgroup Number to undergo research procedures Number to undergo screening procedures

View Young adults with hazardous drinking 870 6000

3.12 Provide a statistical justification for the total number of subjects to be enrolled into this research study at the multicenter sites or this site.

* Described below:

For power consideration, we focus on detecting the difference between EA+I (Arm 1) and Control (Arm 3) in binge drinking days (BDD), since it is the primary comparison of interest. Information from our pilot study was used to estimate the rate of enrollment in the study, the variation in binge episodes up to 3-months and the potential failure to complete the 3-month web-based assessments. If we assume the BDD in EA+I group will decrease by a mean of 3.4 (SD 5.4) from baseline to 3-months follow-up compared to a decrease of 1.1 (SD 4.1) in Control group as we observed in our pilot study, using a sample size ratio of 2:1, we will need 96 participants in EA+I and 48 in Control group to have 80% power to show a difference at significant level = 0.05 based on two-sided two-sample t-test. Including 48 participants in EA group to follow our planned sample ratio 2:1:1, we will need 192 subjects in total. Allowing for a 25% attrition rate, we will actually need to enroll 256 participants in order to achieve a target sample size of 192, divided into 96 EAI, 48 EA and 48 Control participants. If we consider BDD at 9 months, assuming the BDD change will decrease by 35% at 12 months, we need 750 subjects allowing for 30% attrition.

[reviewer notes¬]

Section 3.0 - Human Subjects

3.13 Inclusion Criteria: List the specific criteria for inclusion of potential subjects.

Study participants are eligible if they are 18-25 years of age, screen positive for hazardous dirnking (AUDIT-C), have past month drinking, and personally own a cellular phone with text-message features.

3.14 Exclusion Criteria: List the specific criteria for exclusion of potential subjects from participation.

Ineligibility includes being previously identified or treated for alcohol or substance use disorders, currently treated for psychiatric disease and not speaking English.

3.15 Will HIV serostatus be evaluated specifically for the purpose of participation in this research study?

* no

If Yes, provide a justification:

Section: Section 4 - Recruitment and Informed Consent Procedures

[reviewer notes¬]

Section 4 - Subject Recruitment and Informed Consent Procedures

4.1 Select all recruitment methods to be used to identify potential subjects:

Other Strategies: Described below

Advertisements

Upload the advertisements for review:Name Modified Date

Honest Broker

Identify the name of the honest broker system and name of the specific individuals who will provide those services:

Specify the IRB-assigned honest broker system number (e.g., HB123456):

Upload the signed honest broker assurance agreement:

Name Modified Date

There are no items to display

Recruitment Letters and Scripts

Upload recruitment letters/scripts/text:

Name Modified Date

Research Registry

List the IRB approval number and title for each registry source:

4.2 Provide a detailed description of your recruitment methods, including identifying and initiating contact with participants:

Patients that present to the ED for any illness or injury will be considered for screening if an investigator is available. Patients intake age and acuity will be reviewed by the investigator, and only patients between ages 18-25 and with an Emergency Severity Index (ESI) of 4 or 5 will be considered for enrollment. After the ED care provider determines capacity to provide informed consent, eligible patients will be asked by the ED care provider if they would be interested in learning about the study. An investigator will be stationed in the ED and will be available if the patient indicates to the ED care provider that they would like to hear about the research. Those interested will be approached by an investigator to describe the study.

[reviewer notes¬]

Section 4 - Subject Recruitment and Informed Consent Procedures

4.6 Are you requesting a waiver to document informed consent for any or all participants, for any or all procedures? (e.g., a verbal or computerized consent script will be used, but the subjects will not be required to sign a written informed consent document. This is not a waiver to obtain consent.

* yes

4.6.1 Identify the specific research procedures and/or the specific subject populations for which you are requesting a waiver of the requirement to obtain a signed consent form.

Addressed below:

If not all, identify the specific procedures and/or subject populations for which you are requesting a waiver:

We request a waiver to document written informed consent for screening and for participation in focus groups.

4.6.2 Indicate which of the following regulatory criteria is applicable to your request for a waiver of the requirement to obtain a signed consent form.

45 CFR 46.117(c)(2)

45 CFR 46.117(c)(1) That the only record linking the subject and the research would be the consent document and the principal risk would be potential harm resulting from a breach of confidentiality. Each subject will be asked whether the subject wants documentation linking the subject with the research, and the subject's wishes will govern; or

45 CFR 46.117(c)(2) That the research presents no more than minimal risk of harm to subjects and involves no procedures for which written consent is normally required outside of the research context.

4.6.2.1 Address why the specific research procedures for which you are requesting a waiver of the requirement to obtain a signed consent form present no more than minimal risk of harm to the research subjects:

For screening procedures we are asking questions rotinely asked in clinical care, and part of the CDC's Youth RIsk Behavior Survey, administered annually across the nation.

Focus group participation has no added risk for participants.

4.6.2.2 Justify why the research listed in 4.6.1 involves no procedures for which written informed consent is normally required outside of the research context:

There are no personal identifiers as part of the questions.

Focus group participation is voluntary, and responses will be kept confidential.

4.6.3 Address the procedures that will be used and the information that will be provided (i.e., script) in obtaining and documenting the subjects' verbal informed consent for study participation:

Script as follows:

Thank you for allowing me to talk to you about our research study. My name is [X] and I am a researcher at [Y]. We are conducting a research study over the next year to understand the health behavior among 18-25 year old patients that present to the Emergency Department. As part of our study, we will be asking patients to complete 17 questions about their health, including information about alcohol habits. All information you provide will be kept confidential. We are conducting this research to identify risky health behaviors and will be asking those who report hazardous alcohol use if they would be interested in an interventional research study to test whether a text-message intervention improves health behaviors.

If you qualify for the interventional study based on your initial responses, and you meet our inclusion and exclusion criteria and complete the written informed consent process, we will ask you to complete a contact sheet, and your responses from the initial survey may be linked to other information using the unique ID we give you. There is a risk of a breach in confidentiality if someone not involved in your care gets access to your research records. If you have any questions about the survey, I will be available to assist you. Your participation is voluntary and your participation will not affect your care in the ED.

Do you think you might be interested in participating in this study?

{If No}: Thank you for allowing me to talk with you.

{If Yes}: What I would like you to do is to have you complete a series of questions on this web-based survey instrument. It should take only 5 minutes to complete. There is a possibility that some of the questions may make you uncomfortable; if so, please let me know. You don’t have to answer those questions if you don’t want to. All information you provide will be strictly confidential and will be kept under a password-protected file with only investigators having access.

Upload Scripts:

Name Modified Date

Focus Group Script 9/29/2014 10:45 AM

[reviewer notes¬]

Section 4 - Subject Recruitment and Informed Consent Procedures

4.7 Are you requesting a waiver to obtain informed consent or an alteration of the informed consent process for any of the following?

*no

4.7.1 If Yes, select the reason(s) for your request:

There are no items to display

General Requirements: The Federal Policy [45 CFR 46.116 (d)] specifies in order for a waiver of consent to be approved, the request must meet four criteria. For each request, you will be asked to provide a justification addressing how each of these criterion is met.

Medical record review for the identification of potential subjects:

The research involves no more than minimal risk to the subjects;

[45 CFR 46.116 (d)(1)]

The waiver or alteration will not adversely affect the rights and welfare of the subjects;

[45 CFR 46.116 (d)(2)]

The research could not practicably be carried out without the waiver or alteration;

[45 CFR 46.116 (d)(3)]

Whenever appropriate, the subjects will be provided with additional pertinent information after participation;

[45 CFR 46.116 (d)(4)]

Review of identifiable medical records: [Note: A waiver of HIPAA Authorization must be requested (2.14.2)] Include the approximate number of medical records and/or specimens that will be accessed and enter -1 in question 3.11 for the number of subjects to be enrolled.

The research involves no more than minimal risk to the subjects;

[45 CFR 46.116 (d)(1)]

The waiver or alteration will not adversely affect the rights and welfare of the subjects;

[45 CFR 46.116 (d)(2)]

The research could not practicably be carried out without the waiver or alteration;

[45 CFR 46.116 (d)(3)]

Whenever appropriate, the subjects will be provided with additional pertinent information after participation.

[45 CFR 46.116 (d)(4)]

Parental Permission and/or Child Assent

The research involves no more than minimal risk to the subjects;

[45 CFR 46.116 (d)(1)]

The waiver or alteration will not adversely affect the rights and welfare of the subjects;

[45 CFR 46.116 (d)(2)]

The research could not practicably be carried out without the waiver or alteration;

[45 CFR 46.116 (d)(3)]

Whenever appropriate, the subjects will be provided with additional pertinent information after participation.

[45 CFR 46.116 (d)(4)]

Alteration of informed consent process

The research involves no more than minimal risk to the subjects;

[45 CFR 46.116 (d)(1)]

The waiver or alteration will not adversely affect the rights and welfare of the subjects;

[45 CFR 46.116 (d)(2)]

The research could not practicably be carried out without the waiver or alteration;

[45 CFR 46.116 (d)(3)]

aWhenever appropriate, the subjects will be provided with additional pertinent information after participation.

[45 CFR 46.116 (d)(4)].

Other Minimal Risk activity

The research involves no more than minimal risk to the subjects;

[45 CFR 46.116 (d)(1)]

The waiver or alteration will not adversely affect the rights and welfare of the subjects;

[45 CFR 46.116 (d)(2)]

The research could not practicably be carried out without the waiver or alteration;

[45 CFR 46.116 (d)(3)]

Whenever appropriate, the subjects will be provided with additional pertinent information after participation.

[45 CFR 46.116 (d)(4)].

4.7.2 Under what circumstances (if any) will you obtain consent from some of these subjects?

[reviewer notes¬]

Section 4 - Subject Recruitment and Informed Consent Procedures

4.8 Are you requesting an exception to the requirement to obtain informed consent for research involving the evaluation of an 'emergency' procedure?

Note: This exception allows research on life-threatening conditions for which available treatments are unproven or unsatisfactory and where it is not possible to obtain informed consent.

* no

[reviewer notes¬]

Section 4 - Subject Recruitment and Informed Consent Procedures

4.9 Upload all written informed consent documents.

The FDA requires a new element of consent for “applicable” clinical trials: These are clinical trials registered on clinicaltrials.gov AND are conducted under a U.S. IND or are otherwise subject to FDA regulations.

Click here for more information.

The button is only to be used to upload a new consent form.

Click on the button next to the form name to upload a revised consent form.

Draft Consent Forms for editing:

Name Modified Date

SUFFOLETTO TRAC_Consent_08302013.docx 9/11/2013 8:53 AM

Approved Consent Form(s):

Name Modified Date

SUFFOLETTO TRAC_Consent_08302013.docx 9/11/2013 8:53 AM

[reviewer notes¬]

Section 4 - Subject Recruitment and Informed Consent Procedures

4.10 Will all potential adult subjects be capable of providing direct consent for study participation?

*

Yes

Indicate why direct consent is not possible:

4.10.1 Provide a justification for the inclusion of adult subjects who are unable to provide direct consent for study participation.

4.10.2 Specify the criteria used to determine that a potential adult subject is not able to provide direct consent for participation and identify who will be responsible for that determination.

4.10.3 Will you obtain the potential adult subject's assent for study participation?

*

If No, provide a justification for not obtaining assent:

4.10.4 Identify who will provide proxy consent for the participation of the decisionally impaired adult:

[reviewer notes¬]

Section 4 - Subject Recruitment and Informed Consent Procedures

4.11 At what point will you obtain the informed consent of potential research subjects or their authorized representative?

After performing certain of the screening procedures, but prior to performing any of the research interventions/interactions

If Other, address below:

4.11.1 Address why you feel that it is acceptable to defer obtaining written informed consent until after the screening procedures have been performed.

Obtiaing written consent for screening puts participants at greater risk for breach of confidentiality, being that the written infrmed consent will be the sole document linking their person to the research.

4.11.2 Taking into account the nature of the study and subject population, indicate how the research team will ensure that subjects have sufficient time to decide whether to participate in this study. In addition, describe the steps that will be taken to minimize the possibility of coercion or undue influence.

Potential subjects will be told to please take their time in deciding whether they want to be involved in the clinical trial; as well, they will be encouraged to discuss the decision with friends and family or with the health care team. The investigator will describe the study and leave the room to allow for adequate reflection time and return several minutes later. Patients will be told that their participation or non-participation will not influence their care or relationship with care providers.

[reviewer notes¬]

Section 4 - Subject Recruitment and Informed Consent Procedures

4.12 Describe the process that you will employ to ensure the subjects are fully informed about this research study.

* Addressed below:

This description must include the following elements:

who from the research team will be involved in the consent process (both the discussion and documentation);

person who will provide consent or permission;

information communicated; and

any waiting period between informing the prospective participant about the study and obtaining consent

An investigator will explain to potential participants the details of what is involved in the clinical trial. They will go over the informed consent document to help the patient understand what will happen in the study, why the study is being done, what the study investigators hope to accomplish, and what risks or benefits might be involved in the study. Potential participants will be told to please take their time in deciding whether they want to be involved in the clinical trial; as well, they will be encouraged to discuss the decision with friends and family or with the health care team. The investigator will leave the room to allow for adequate reflection time and return several minutes later.

4.13 Are you requesting an exception to either IRB policy related to the informed consent process?

For studies involving a drug, device or surgical procedures, a listed physician investigator is required to obtain the written informed consent unless an exception to this policy has been approved by the IRB

For all other studies, a listed investigator is required to obtain consent (Note: In order to request an exception to this policy, the study must be minimal risk)

* no

If Yes, provide a justification and describe the qualifications of the individual who will obtain consent:

4.14 Will you inform research subjects about the outcome of this research study following its completion?

* no

If Yes, describe the process to inform subjects of the results:

Section: Section 5 - Potential Risks and Benefits

[reviewer notes¬]

Section 5 - Potential Risks and Benefits of Study Participation

5.1 Describe potential risks (physical, psychological, social, legal, economic or other) associated with screening procedures, research interventions/interactions, and follow-up/monitoring procedures performed specifically for this study:

* View Research Activity: Research Web-based Assessments

Common Risks: No Value Entered

Infrequent Risks: Discomfort with answering questions of a sensitive nature

Other Risks: Possible breach of confidentiality

View Research Activity: Screening

Common Risks: No Value Entered

Infrequent Risks: Discomfort with answering questions of a sensitive nature

Other Risks: No Value Entered

View Research Activity: Text message dialogue

Common Risks: No Value Entered

Infrequent Risks: No Value Entered

Other Risks: Possible breach of confidentiality

5.1.1 Describe the steps that will be taken to prevent or to minimize the severity of the potential risks:

We will make every effort to ensure that subject information is protected so only authorized persons can see their information. For data entered via our web-site, we will only link participant information to their ID and all data will be stored PER UPMC standards. For SMS dialogue, all messages sent from subjects to our phone number will be catalogued and encrypted, stored per UPMC security standards. We state explicitly in the consent that we will not be able to respond in real-time to any concern a patient has that is outside the expected responses that is sent to our phone number, nor any text message outside the scope of the question we ask. Immediate or emergency text-messages that are recieved and outside the scope of the questions we sent will all recieve a standardized text message, stating "We appreciate your participation. If you have an emergency, please call 911." As well, all messages are archived in our databse and will be periodically reviewed by the investigators.

5.2 What steps will be taken in the event that a clinically significant, unexpected disease or condition is identified during the conduct of the study?

* Not Applicable

5.3 All the risk questions (screening, intervention/interaction, follow-up) have been merged into one question (5.1).

[reviewer notes¬]

Section 5 - Potential Risks and Benefits of Study Participation

5.4 Do any of the research procedures pose a physical or clinically significant psychological risk to women who are or may be pregnant or to a fetus?

* no

5.4.1 List the research procedures that pose a risk to pregnant women or fetuses:

5.4.2 Describe the steps that will be taken to rule out pregnancy prior to exposing women of child-bearing potential to the research procedures that pose a risk to pregnant women or fetuses:

5.4.3 Describe the measures to prevent pregnancy, and their required duration of use, that will be discussed with women of child-bearing potential during and following exposure to research procedures:

[reviewer notes¬]

Section 5 - Potential Risks and Benefits of Study Participation

5.5 Do any of the research procedures pose a potential risk of causing genetic mutations that could lead to birth defects?

* No

5.5.1 List the research procedures that pose a potential risk of genetic mutations/birth defects:

5.5.2 Describe the measures to prevent pregnancy, and their required duration of use, in female subjects and female partners of male subjects during and following exposure to research procedures:

[reviewer notes¬]

Section 5 - Potential Risks and Benefits of Study Participation

5.6 Are there any alternative procedures or courses of treatment which may be of benefit to the subject if they choose not to participate in this study?

* No

If Yes, describe in detail:

[reviewer notes¬]

Section 5 - Potential Risks and Benefits of Study Participation

5.7 Describe the specific endpoints (e.g., adverse reactions/events, failure to demonstrate effectiveness, disease progression) or other circumstances (e.g., subject's failure to follow study procedures) that will result in discontinuing a subject’s participation?

* Not applicable - There are no anticipated circumstances that would lead to discontinuing a subject’s participation in this research study.

[reviewer notes¬]

Section 5 - Potential Risks and Benefits of Study Participation

5.8 Will any individuals other than the investigators/research staff involved in the conduct of this research study and authorized representatives of the University Research Conduct and Compliance Office (RCCO) be permitted access to research data/documents (including medical record information) associated with the conduct of this research study?

* no

5.8.1 Identify the 'external' persons or entity who may have access to research data/documents and the purpose of this access:

5.8.2 Will these 'external' persons or entity have access to identifiable research data/documents?

*

If Yes, describe how they will protect the confidentiality of the research data:

5.9 Has or will a Federal Certificate of Confidentiality be obtained for this research study?

* yes

5.10 Question has been moved to 5.17

5.11 Question has been moved to 5.16

[reviewer notes¬]

Section 5 - Potential Risks and Benefits of Study Participation

5.12 Does participation in this research study offer the potential for direct benefit to the research subjects?

No - Describe the general benefits to society (e.g., increased knowledge; improved safety; better health; technological advancement) that may result from the conduct of this research study.

Describe the benefit:

Participating in this study may help determine whether mobile phone text-messaging can be used to reduce hazardous alcohol use among young adults.

5.13 Describe the data and safety monitoring plan associated with this study. If the research study involves multiple sites, the plan must address both a local and central review process.

Study progress, recruitment, confidentiality, data quality and analysis will be reviewed by the investigators at departmental research meetings (approximately once per month) and written records will be kept by the investogators. Reports will be submitted to the IRB at time of annual renewal. Monitoring will comply with the IRB policy for reporting adverse events at outlined in Chapter 3.0 of the IRB Reference Manual.

[reviewer notes¬]

Section 5 - Potential Risks and Benefits of Study Participation

5.14 What precautions will be used to ensure subject privacy is respected? (e.g. the research intervention will be conducted in a private room; the collection of sensitive information about subjects is limited to the amount necessary to achieve the aims of the research, so that no unneeded sensitive information is being collected, drapes or other barriers will be used for subjects who are required to disrobe)

All questions and conversations will take place in a private room. We are only collecting information necessary to correctly interpret results of our study. Our study is limited to information regarding alcohol intake and other related risk factors.

5.15 What precautions will be used to maintain the confidentiality of identifiable information? (e.g., paper-based records will be kept in a secure location and only be accessible to personnel involved in the study, computer-based files will only be made available to personnel involved in the study through the use of access privileges and passwords, prior to access to any study-related information, personnel will be required to sign statements agreeing to protect the security and confidentiality of identifiable information, whenever feasible, identifiers will be removed from study-related information, precautions are in place to ensure the data is secure by using passwords and encryption, because the research involves web-based surveys, audio and/or video recordings of subjects will be transcribed and then destroyed to eliminate audible identification of subjects)

All paper-based records collected in the ED will be sealed in an opaque envelope and stored in a secure location in the offices of Emergency Medicine. All computer-based files from mobile phone text messaging will only be made available to personnel involved in the study through the use of access privileges and passwords. Prior to access to any study-related information, personnel will be required to sign statements agreeing to protect the security and confidentiality of identifiable information, whenever feasible, identifiers will be removed from study-related information. Precautions are in place to ensure the data is secure by using passwords and encryption, because the research involves web-based surveys and mobile phone text messages.

5.16 If the subject withdraws from the study, describe what, if anything, will happen to the subject’s research data or biological specimens.

If a subject withdraws consent, any identifiable research or medical information recorded for, or resulting from, their participation in this research study prior to the time they formally withdraw consent may continue to be used and disclosed by investigators for the purposes described above.

5.17 Following the required data retention period, describe the procedures utilized to protect subject confidentiality. (e.g., destruction of research records; removal of identifiers; destruction of linkage code information; secured long-term retention)

The investigators may continue to use and disclose, for the purposes described above, identifiable information (which may include identifiable medical information) related to participation in this research study for a minimum of seven years after final reporting or publication of a project. Following this time period, all materials, printed or digital, will be destroyed, including linkage codes.

Section: Section 6 - Costs and Payments

[reviewer notes¬]

Section 6 - Costs and Payments

6.1 Will research subjects or their insurance providers be charged for any of the procedures (e.g., screening procedures, research procedures, follow-up procedures) performed for the purpose of this research study?

*

No

6.1.1 Specify what research procedures will be billed to the subject or insurance provider:

6.1.2 Provide a justification for billing subjects or insurance providers for procedures that are performed solely for the purpose of the research study.

6.1.3 Will subjects or insurance providers be billed for the investigational drug or device being evaluated or used in this research study?

*

Provide assurance that the FDA has given approval for the sponsor of this research study to charge investigators for the investigational drug or device.

If this is an investigational device, indicate if the Health Care Financing Administration has designated it as a Class B medical device.

*

6.1.4 Address the contingencies that are in place to ensure that potential subjects, who may desire to participate in this research study but are not able to bear this personal financial risk, will be afforded access to study participation.

[reviewer notes¬]

Section 6 - Costs and Payments

6.2 Will subjects be compensated in any way for their participation in this research study?

* yes

6.2.1 Describe the amount of payment or other remuneration offered for complete participation in this research study.

They will be paid a total of $100 if they complete all parts of this study up to 9-month web-based follow-up.

For participants who complete the focus group, they will be paid an additional $60.00.

6.2.2 Describe the amount and term of payment or other remuneration that will be provided for partial completion of this research study.

The cost of text messaging will be covered in the flat fees for reimbursement for participation. If, for whatever reason, they complete part but not all of the study, the terms of this payment will be as follows: 1) $10 for completing the initial questionnaires AND $20.00 for 3 month web-based assessment, $30.00 for 6-month web-based assessments and $40.00 for completion of 9-month web-based assessments.

Section: Section 7 - Qualifications and Source(s) of Support

[reviewer notes¬]

Section 7 - Qualifications of Investigators and Sources of Research Study

7.1 Summarize the qualifications and expertise of the principal investigator and listed co-investigators to perform the procedures outlined in this research study.

Brian Suffoletto, MD MS, is an experienced clinical researcher and a staff physician at UPMC Mercy. He has experience screening patients for risky alcohol use, enrolling adults in clinical trials and following up after ED discahrge. He has attended and presented at DSMBs as well.

Jeffrey Kristan is a research assistant who has extensive experience screening young adults for risky drinking and has completed all regulatory documents and training.

Sandra Truong and Sydney Huerbin are trained research assistants who has extensive familiarity with humans subjects research, including 2 years screening and enrolling young adults in clinical trials.

Duncan Clark, MD PhD, is the Director of the Pittsburgh Adolescent Alcohol Research Center (PAARC) at Western Psychiatry Institiute of UPMC, and is an experienced drug and alcohol counselor and behavioral specialist.

Laurel Person Mecca from the University Center for Social and Urban Research will assist with conducting the focus group sessions. She is a highly trained individual in this field.

Andrew Abboud, Naadia Ahmed, Paula Amin, Ryan Begun, Nisha Bhat, Neeti Bhatte, Victoria Bianco, Megan Buhay, Patrick Coppler, Melissa Delia, Kathryn Ferruzza, Danyelle Fraser, Helen Gaim, Erin Gilchrist, Kylie Grady, Alec Hawley, Rachael Hellman, Brian Killeen, Janera Koehler, Jonathan Korpon, Kyle McManigle, Shane Mealy, Jeff Moorhead, Mitchell Moyer, Ayodele Ogunmola, Evan Reinhart, Margaret Russell, Jordan Siegel are all MACRO students and have completed all requisite training.

[reviewer notes¬]

Section 7 - Qualifications of Investigators and Sources of Study Support

7.2 Indicate all sources of support for this research study.

*

Selections

Foundation: Upload a copy of the research plan that was submitted to the agency.

If Federal support, provide the sponsor information:

Federal sponsor Grant Title Grant number Awardee institution Federal grant application

For projects not supported by a federal grant, upload the research plan that was submitted for funding:

Name Modified Date

EMF_final.docx 8/21/2012 10:22 AM

If Industry support, provide the sponsor information and level of support:

If Foundation support, provide the sponsor information:

Emergency Medicine Foundation (EMF)

If Other support, provide the support information and level of support:

[reviewer notes¬]

Section 7 - Qualifications of Investigators and Sources of Research Study

7.3 Is this study funded in part or whole by a PHS Agency?

* no

Does any investigator* involved in this study (select all that apply):

Name

A. Have a financial interest (aggregated value of equity and remuneration** during the past or next twelve months) in a publicly-traded entity that either sponsors*** this research or owns the technology being evaluated or developed that exceeds $5,000 but not $10,000?

B. Have a financial interest (aggregated value of equity and remuneration during the past or next twelve months) in a publicly-traded entity that either sponsors this research or owns the technology being evaluated or developed that exceeds $10,000?

C. Receive remuneration (during the past or next twelve months) from a non-publicly traded entity that either sponsors this research or owns the technology being evaluated or developed that exceeds $5,000 but not $10,000?

D. Receive remuneration (during the past or next twelve months) from a non-publicly traded entity that either sponsors this research or owns the technology being evaluated or developed that exceeds $10,000?

E. Have equity in a non-publicly traded entity that either sponsors this research or owns the technology being evaluated or developed?

F. Receive reimbursement or sponsorship of travel expenses (for one trip or a series of trips during the past or next twelve months) by an outside entity that either sponsors this research or owns the technology being evaluated or developed that exceeds $5,000?

G. Have rights as either the author or inventor of intellectual property being evaluated or developed in this research that is the subject of an issued patent or has been optioned or licensed to an entity?

H. Have an officer or management position**** with a Licensed Start-up Company overseen by the COI Committee that either sponsors this research or owns the technology being evaluated or developed?

I. Receive compensation of any amount when the value of the compensation would be affected by the outcome of this research, such as compensation that is explicitly greater for a favorable outcome than for an unfavorable outcome or compensation in the form of an equity interest in the entity that either sponsors this research or owns the technology being evaluated or developed?

None of the above options apply and there are no other financial conflicts of interest in the conduct of this research.

*Investigator means the PI, co-investigators, and any other member of the study team, regardless of title, who participates in the design, conduct, or reporting of this research, as well as his/her spouse, registered domestic partner, dependents, or other members of his/her household. The PI is responsible for ensuring that s/he and all other relevant members of the study team review the above questions describing Significant Financial Interests.

**such as salary, consulting fees, honoraria, or paid authorship

***through the provision of funds, drugs, devices, or other support for this research

****Such as serving on the Board of Directors or Board of Managers or a position that carries a fiduciary responsibility to the company (e.g., CEO, CFO, CTO, or CMO).

Does any investigator* involved in this study (select all that apply):

Name

A. Have equity in a publicly-traded entity that either sponsors** this research or owns the technology being evaluated or developed that exceeds a 5% ownership interest or a current value of $10,000?

B. Have equity in a non-publicly-traded entity that either sponsors this research or owns the technology being evaluated or developed?

C. Receive salary, consulting fees, honoraria, royalties or other remuneration from an entity that either sponsors this research or owns the technology being evaluated or developed that is expected to exceed $10,000 during the past or next 12 months?

D. Have rights as either the author or inventor of intellectual property being evaluated or developed in this research that is the subject of an issued patent or has been optioned or licensed to an entity?

E. Have an officer or management position**** with a Licensed Start-up Company overseen by the COI Committee that either sponsors this research or owns the technology being evaluated or developed?

F. Receive compensation of any amount when the value of the compensation would be affected by the outcome of this research, such as compensation that is explicitly greater for a favorable outcome than for an unfavorable outcome or compensation in the form of an equity interest in the entity that either sponsors this research or owns the technology being evaluated or developed?

None of the above options apply and there are no other financial conflicts of interest in the conduct of this research.

*Investigator means the PI, co-investigators, and any other member of the study team, regardless of title, who participates in the design, conduct, or reporting of this research, as well as his/her spouse, registered domestic partner, dependents, or other members of his/her household. The PI is responsible for ensuring that s/he and all other relevant members of the study team review the above questions describing Significant Financial Interests.

**through the provision of funds, drugs, devices, or other support for this research

****Such as serving on the Board of Directors or Board of Managers or a position that carries a fiduciary responsibility to the company (e.g., CEO, CFO, CTO, or CMO).

7.3.1 Provide the name of the investigator(s) and describe the nature of the Significant Financial Interest(s):

7.3.2 If you selected A, B, C, or E, please complete a Standard Conflict of Interest Management Plan and submit it with your protocol. Please provide all of the requested information, including the correct protocol number and title. Incomplete, inaccurate, or unsigned forms will have to be edited and replaced.

For all other financial interests (D or F), the COI Office will work with you to develop an appropriate COI Management Plan.

7.3.2 If you selected B, D, E, or H, please complete a Standard Conflict of Interest Management Plan and submit it with your protocol. Please provide all of the requested information, including the correct protocol number and title. Incomplete, inaccurate, or unsigned forms will have to be edited and replaced.

For all other financial interests (A, C, F, or G), the COI Office will work with you to develop an appropriate COI Management Plan.

*

Name Modified Date
